# Sex Differences in Delayed Hospitalization in Patients with Non-ST-Segment Elevation Myocardial Infarction Undergoing New-Generation Drug-Eluting Stent Implantation

**DOI:** 10.3390/jcm12051982

**Published:** 2023-03-02

**Authors:** Yong Hoon Kim, Ae-Young Her, Seung-Woon Rha, Cheol Ung Choi, Byoung Geol Choi, Ji Bak Kim, Soohyung Park, Dong Oh Kang, Ji Young Park, Woong Gil Choi, Sang-Ho Park, Myung Ho Jeong

**Affiliations:** 1 Division of Cardiology, Department of Internal Medicine, Kangwon National University School of Medicine, Chuncheon 24289, Republic of Korea; 2 Cardiovascular Center, Korea University Guro Hospital, Seoul 08308, Republic of Korea; 3 Cardiovascular Research Institute, Korea University College of Medicine, Seoul 02841, Republic of Korea; 4 Division of Cardiology, Department of Internal Medicine, Cardiovascular Center, Nowon Eulji Medical Center, Eulji University, Seoul 01830, Republic of Korea; 5 Division of Cardiology, Department of Internal Medicine, Chungbuk National University Hospital, College of Medicine, Chungbuk National University, Cheongju 28644, Republic of Korea; 6 Cardiology Department, Soonchunhyang University Cheonan Hospital, Cheonan 31151, Republic of Korea; 7 Department of Cardiology, Cardiovascular Center, Chonnam National University Hospital, Gwangju 61469, Republic of Korea

**Keywords:** non-ST-elevation myocardial infarction, sex discrepancies, prehospital delay

## Abstract

We compared the effects of sex differences in delayed hospitalization (symptom-to-door time [SDT], ≥24 h) on major clinical outcomes in patients with non-ST-segment elevation myocardial infarction after new-generation drug-eluting stent implantation. A total of 4593 patients were classified into groups with (*n* = 1276) and without delayed hospitalization (SDT < 24 h, *n* = 3317). Thereafter, these two groups were subdivided into male and female groups. The primary clinical outcomes were major adverse cardiac and cerebrovascular events (MACCE), defined as all-cause death, recurrent myocardial infarction, repeat coronary revascularization, and stroke. The secondary clinical outcome was stent thrombosis. After multivariable- and propensity score-adjusted analyses, in-hospital mortalities were similar between the male and female groups in both the SDT < 24 h and SDT ≥ 24 h groups. However, during a 3-year follow-up period, in the SDT < 24 h group, all-cause death (*p* = 0.013 and *p* = 0.005, respectively) and cardiac death (CD, *p* = 0.015 and *p* = 0.008, respectively) rates were significantly higher in the female group than those in the male group. This may be related to the lower all-cause death and CD rates (*p* = 0.022 and *p* = 0.012, respectively) in the SDT < 24 h group than in the SDT ≥ 24 h group among male patients. Other outcomes were similar between the male and female groups and between the SDT < 24 h and SDT ≥ 24 h groups. In this prospective cohort study, female patients showed higher 3-year mortality, especially in the SDT < 24 h, compared to male patients.

## 1. Introduction

Acute myocardial infarction (AMI) occurs due to thrombus formation resulting from a rupture or erosion of vulnerable atherosclerotic plaques [1]. The contributing risk factors for plaque instability include atherosclerotic risk factors and other diverse factors [2,3]. The sudden imbalance between myocardial oxygen consumption and demand is also an important cause of myocardial infarction and is caused by coronary artery spasm, coronary embolism, coronary arteritis, anemia, hypotension, tachycardia, hypertrophic cardiomyopathy, and severe aortic stenosis [4]. Females have different characteristics compared to men. For example, they generally have smaller body surface area and smaller coronary arteries, and they have various sex hormone-mediated factors [5]. Previous [6] and recent [7] reports showed that females presenting with acute myocardial infarction had higher mortality rates than males. Many other results between the male and female sexes after acute coronary syndrome (ACS) [8,9] were not consistent, and definite causative factors contributing to poorer clinical outcomes in females have not been definitely identified. Old age and increased incidence of diabetes mellitus (DM), chronic heart failure (HF), hypertension prior to MI [10], underestimation of cardiovascular risk for females [11], and delayed presentation with more atypical symptoms were suggested for the explanation of poorer results in females [12]. One report showed that atypical symptoms were associated with pre-hospital delay in 1894 in patients with acute ACS [13]. In patients with ST-segment elevation myocardial infarction (STEMI), rapid reperfusion of the infarct-related artery (IRA) reduces the mortality rate, and the maximum time delay from STEMI diagnosis to reperfusion of the IRA is 120 min [14]. Recent research [15] showed that long-term mortality is strongly related to total ischemic time rather than door-to-balloon time (DBT), because DTB has reached its limit of effect. In patients with non-STEMI (NSTEMI), the current guideline [16] recommends that an early coronary angiography [CAG] and percutaneous coronary intervention [PCI] within 24 h of admission (early invasive) is preferred over a delayed invasive strategy that includes at least one high-risk criterion. However, the optimal timing of PCI in NSTEMI is debatable [17] and is yet to be fully evaluated. Similar to the findings of Eggers’s study [17], one study [18] showed that the major clinical outcomes were not significantly different between the early invasive and delayed invasive groups in patients with NSTEMI and complex lesions after new-generation DES implantation. However, Fox et al. [19] suggested that a routine invasive strategy reduces long-term rates of cardiovascular death or MI, and the largest absolute effect is seen in higher-risk patients. Furthermore, only a few studies [20,21] have investigated the long-term clinical outcomes in patients who had delayed hospitalization (symptom-to-door time [SDT] ≥ 24 h). Therefore, in this study, we compared the 3-year effects of sex differences in delayed hospitalization on major clinical outcomes in patients with NSTEMI after new-generation drug-eluting stent implantation. 

## 2. Methods

### 2.1. Study Population

**Figure 1:**
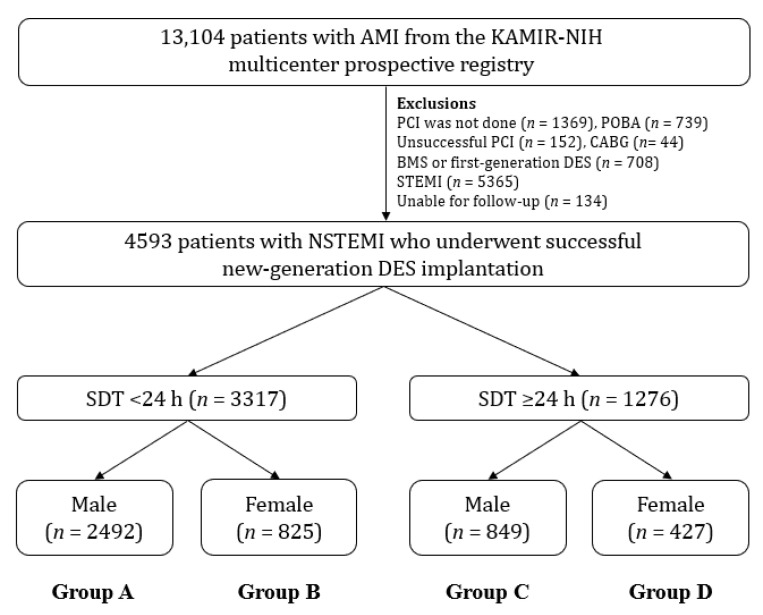
Flowchart. AMI, acute myocardial infarction; KAMIR-NIH, Korea Acute Myocardial Infarction Registry-National Institute of Health; PCI, percutaneous coronary intervention; POBA, plain old balloon angioplasty CABG, coronary artery bypass graft; BMS, bare-metal stent; DES, drug-eluting stent; STEMI, ST-segment-elevation myocardial infarction; NSTEMI, non-STEMI; SDT, symptom-to-door time.

This was a non-randomized, multicenter, prospective cohort study. From the Korea AMI Registry-National Institute of Health (KAMIR-NIH) [22], a total of 13,104 AMI patients were recruited from November 2011 to December 2015. KAMIR-NIH [22] is a nationwide, prospective, multicenter registry comprising 20 high-volume PCI centers in the Republic of Korea. Its website is http://www.kamir.or.kr (accessed on 1 November 2011). At the time of initial enrollment, only patients aged 18 and over were included. The exclusion criteria of this study were as follows: patients who did not undergo PCI (*n* = 1369, 10.4%); those who underwent plain old balloon angioplasty (*n* = 739, 5.6%); unsuccessful PCI (*n* = 152, 1.2%); coronary artery bypass graft (CABG, *n* = 44, 0.3%); or BMS, or first-generation (1G)-DES implantation (*n* = 708, 5.4%); those who had STEMI (*n* = 5365, 40.9%); or those who were lost to follow-up (*n* = 134, 1.0%) (Figure 1). Overall, 4593 patients with NSTEMI who underwent successful PCI using new-generation DES were enrolled and classified into SDT < 24 h (*n* = 3317, 72.2%) and SDT ≥ 24 h (*n* = 1276, 27.8%) groups, and these two groups were subdivided into male (group A [*n* = 2492] and group C [*n* = 849]) and female (group B [*n* = 825] and group D [*n* = 427]) subgroups (Figure 1). We described the types of DES new-generation that were used during the PCI within the footnotes of Table 1. This study was approved by the Ethics Committee of each participating center and the Chonnam National University Hospital Institutional Review Board Ethics Committee (CNUH-2011-172) according to the ethical guidelines of the 2004 Declaration of Helsinki. All 4593 patients included in the study provided written informed consent before enrollment. They finished their 3-year clinical follow-up through face-to-face interviews, phone calls, and chart reviews. From all participating PCI centers, the enrolled data were collected using a web-based system. Event adjudication processes have been described in a previous publication by KAMIR investigators [22]; event adjudication processes have been mentioned.

### 2.2. Percutaneous Coronary Intervention and Medical Treatment

After conventional CAG via a transfemoral or transradial approach [23], 200–300 mg of aspirin, 300–600 mg of clopidogrel, 180 mg of ticagrelor, and 60 mg of prasugrel were prescribed as the loading doses before PCI. After PCI, 100 mg of aspirin was recommended for all patients, combined 75 mg of clopidogrel once daily, 90 mg of ticagrelor twice daily, or 5–10 mg of prasugrel once daily for a minimum of one year. The individual operators were able to choose the access site, revascularization strategy, and DES without any restrictions.

### 2.3. Study Definitions and Clinical Outcomes

Based on current guidelines, NSTEMI was defined as the absence of persistent STE with increased cardiac biomarker levels in an appropriate clinical context [4,24]. In the IRA, successful PCI was defined as <30% residual stenosis and thrombolysis of MI flow grade 3. To obtain more precise results, a Global Registry of Acute Coronary Events (GRACE) risk score [25] was calculated for all study population. Patients with SDT ≥ 24 h were included in the delayed hospitalization group based on the findings of a recent report [20]. In our study, we defined the symptom onset time as the time of onset of the last sustained chest pain of the individual patients [26]. We also defined typical chest pain as substernal chest discomfort of characteristic quality and duration, triggered by exertion or emotional stress, and relieved by rest or nitroglycerin use [24]. Atypical chest pain was defined as chest pain that was inconsistent with the characteristics of typical chest pain. In this study, major adverse cardiac and cerebrovascular events (MACCE), defined as all-cause death, recurrent MI (re-MI), any repeat coronary revascularization, and stroke were considered as the primary clinical outcome. Target lesion revascularization, target vessel revascularization (TVR), and non-TVR were included in the criteria for any repeat revascularization. The event rate of definite or probable stent thrombosis was considered as the secondary clinical outcome. When a definite non-cardiac cause was not approved, all-cause death was considered cardiac death (CD) [27]. In a previous report, we defined the definitions of re-MI, target lesion revascularization, TVR, and non-TVR [28]. We defined the definition of stroke according to the American Heart Association/American Stroke Association guidelines [29], such as an acute cerebrovascular event resulting in death or neurological deficit for >24 h or the presence of acute infarction demonstrated by brain imaging studies. By guidelines suggested by the Academic Research Consortium, ST was defined [30].

### 2.4. Statistical Analyses

We used the SPSS software version 20 (IBM, Armonk, NY, USA) to perform statistical analyses. For continuous variables, intergroup differences were evaluated using the unpaired *t*-test, and data were expressed as mean ± standard deviation or median (interquartile range). For categorical variables, intergroup differences were analyzed using the chi-square or Fisher’s exact test, and data were expressed as counts and percentages. Both in the groups with or without delayed hospitalization, univariate analyses were performed for all variables with the assumption that *p* value at <0.05 is a significant value. Subsequently, a multicollinearity test [31] was performed for the included variables to confirm non-collinearity among them (Table S1). Among the variables, variance inflation factor values were calculated to measure the degree of multicollinearity. A high correlation was suspected when the variance inflation factor value exceeds 5 [32]. Moreover, when the tolerance value was less than 0.1 [33] or the condition index was more than 10 [32], multicollinearity was suspected [33]. In this study, the following variables were included in the multivariable Cox regression analysis; age, left ventricular ejection fraction (LVEF), body mass index, diastolic blood pressure, DBT, cardiogenic shock, cardiopulmonary resuscitation (CPR) on admission, atypical chest pain, dyspnea, Q-wave, ST-segment depression, and T-wave inversion on electrocardiogram; Killip class II/III; non-PCI center; PCI center; hypertension; diabetes mellitus; previous heart failure; previous stroke; current smoker; levels of peak creatine kinase myocardial band (CK-MB); and blood glucose, serum creatinine, total cholesterol, triglyceride, high-density lipoprotein cholesterol, and low-density lipoprotein cholesterol (Table S1). Moreover, to correct the confounding variables, a propensity score (PS)-adjusted analysis was performed using a logistic regression model. All baseline characteristics shown in Table 1 were included in the PS-adjusted analysis. The c-statistic for the PS-matched analysis in this study was 0.710. Using the nearest available pair-matching method in a 1:1 fashion, patients in the SDT ≥ 24 h group were matched to those in the SDT < 24 h group. The used caliper width was 0.01. Table S2 shows baseline characteristics between the male and female groups before and after PS-matched analysis. Various clinical outcomes were estimated using Kaplan–Meier curve analysis and long-rank was used to compare the group differences. A calculated *p* value at <0.05 is considered statistically significant. Table S3 shows the results of the collinearity test for MACCE between the <24 h and ≥24 h groups.

## 3. Results

### 3.1. Baseline Characteristics

Table 1, Tables S2, S4 and S5 show baseline characteristics. In both the SDT < 24 h and SDT ≥ 24 h groups, the mean values of body mass index, mean diameter of deployed stents, number of current smokers, prasugrel as a discharge medication, transradial approach, and use of intravascular ultrasound and optical coherence tomography were higher in the male group than in the female group. In contrast, the mean age, mean value of blood glucose, GRACE risk score, and number of patients with atypical chest pain who showed ST-segment depression and T-wave inversion on EKG, Killip classes II and III, hypertension, diabetes mellitus, previous history of heart failure and stroke, clopidogrel as a discharge medication, the left anterior descending coronary artery as a treated vessel, and a high GRACE risk score (>140) were higher in the female group. Figure S1 shows diverse causes of AMI.

### 3.2. Clinical Outcomes

**Figure 2:**
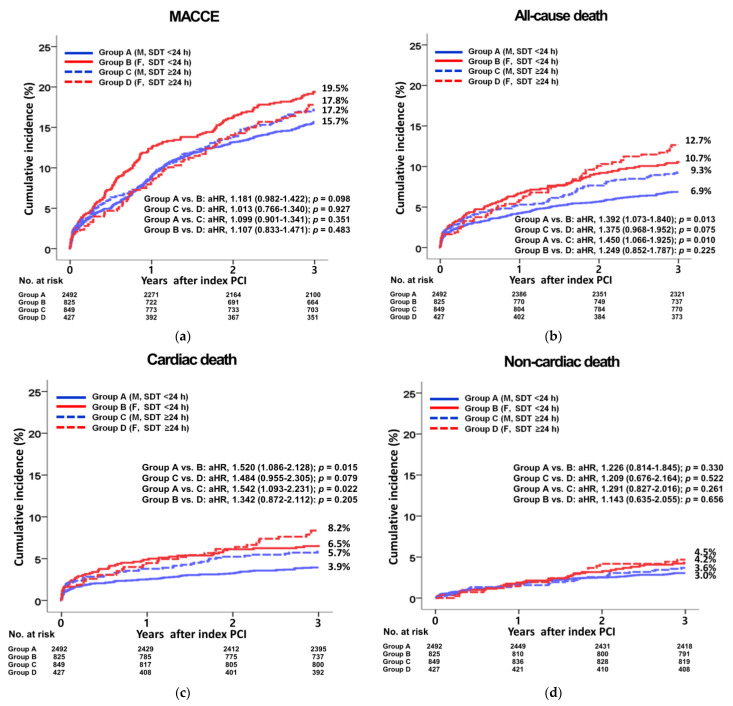

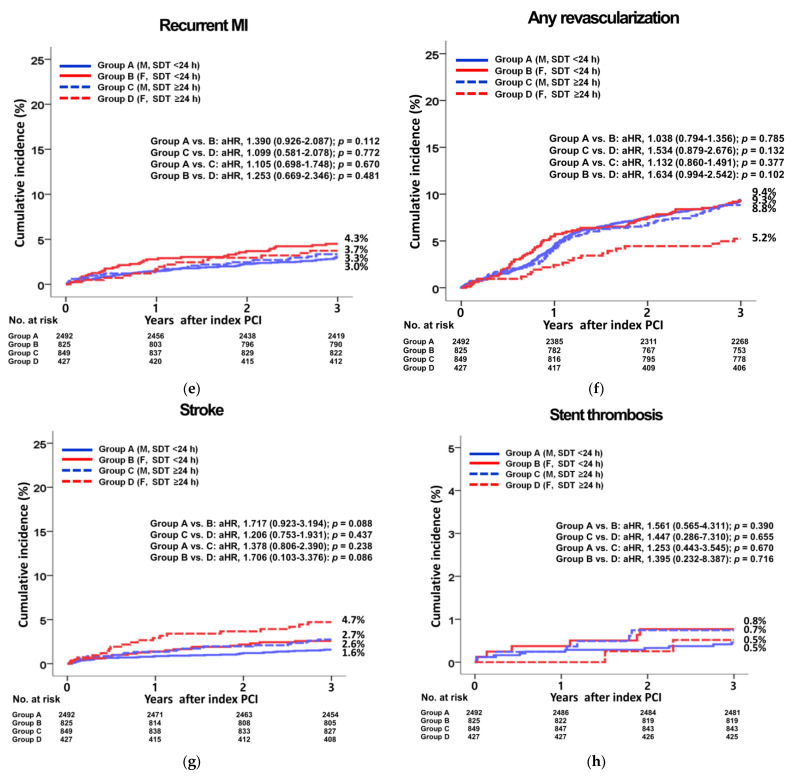
Kaplan–Meier curved analysis for MACCE (**a**), all-cause death (**b**), cardiac death (**c**), non-cardiac death (**d**), recurrent MI (**e**), any repeat revascularization (**f**), stroke (**g**), and stent thrombosis (**h**). MACCE, major adverse cardiac and cerebrovascular events; M, male; F, female; SDT, symptom-to-door time; aHR, adjusted hazard ratio; CI, confidence interval; MI, myocardial infarction.

Table 2 and Table 3, Figure 2a–h show 3-year major clinical outcomes. After multivariable-adjusted analysis, in-hospital all-cause death rates were not significantly different between the male and female groups in both the SDT < 24 h (adjusted hazard ratio [aHR], 1.034; *p* = 0.913) and SDT ≥ 24 h (aHR, 1.218; *p* = 0.707) groups. Similarly, in-hospital CD rates were not significantly different between the male and female groups in both the SDT < 24 h (aHR, 1.324; *p* = 0.420) and SDT ≥ 24 h (aHR, 1.011; *p* = 0.984) groups. These results were confirmed by PS-adjusted analyses. During a 3-year follow-up period, in the < 24 h group, multivariable-adjusted analysis revealed that MACCE (aHR, 1.181; 95% confidence interval [CI], 0.982–1.422; *p* = 0.098), non-CD (NCD, aHR, 1.226; *p* = 0.330), recurrent MI (aHR, 1.390; *p* = 0.112), any repeat revascularization (aHR, 1.038; *p* = 0.785), stroke (aHR, 1.562; *p* = 0.109), and ST (aHR, 1.561; *p* = 0.390) rates were not significantly different between the male and female groups. However, all-cause death (aHR, 1.392; *p* = 0.013) and CD (aHR, 1.520; *p* = 0.015) rates were significantly higher in the female group than in the male group. These results were confirmed by the PS-adjusted analysis. In the SDT ≥ 24 h group, after multivariable-adjusted and PS-adjusted analyses, MACCE (*p* = 0.927 and *p* = 0.561, respectively), all-cause death (*p* = 0.075 and *p* = 0.087, respectively), CD (*p* = 0.079 and *p* = 0.095, respectively), NCD (*p* = 0.522 and *p* = 0.245, respectively), recurrent MI (*p* = 0.772 and *p* = 0.746, respectively), any repeat revascularization (*p* = 0.132 and *p* = 0.101, respectively), stroke (*p* = 0.088 and *p* = 0.198, respectively), and ST (*p* = 0.655 and *p* = 0.758, respectively) rates were not significantly different between the male and female groups. In the total study population, after the multivariable-adjusted and PS-adjusted analyses, all-cause death (*p* = 0.001 and *p* = 0.002, respectively) and CD (*p* = 0.002 and *p* = 0.004, respectively) were significantly higher in the female group than in the male group. In Table 3, after multivariable-adjusted analysis, in-hospital all-cause death rates were not significantly different between the SDT < 24 h and SDT ≥ 24 h groups in both the male (aHR, 1.425; *p* = 0.327) and female (aHR, 1.143; *p* = 0.813) groups. Moreover, in-hospital CD rates were not significantly different between the SDT < 24 h and SDT ≥ 24 h groups in both the male (aHR, 2.036; *p* = 0.101) and SDT ≥ 24 h (aHR, 1.618; *p* = 0.432) groups. These results were confirmed by PS-adjusted analyses. During a 3-year follow-up period in the male group, the multivariable-adjusted analysis revealed that all-cause death (aHR, 1.450; *p* = 0.010) and CD (aHR, 1.542; *p* = 0.022) rates were significantly higher in the SDT ≥ 24 h group than those in the SDT < 24 h group. However, MACCE, NCD, recurrent MI, any repeat revascularization, stroke, and ST rates were not significantly different between the SDT < 24 h and SDT ≥ 24 h groups. These results were confirmed by the PS-adjusted analysis. In the female group, after multivariable-adjusted and PS-adjusted analyses, MACCE (*p* = 0.483 and *p* = 0.385, respectively), all-cause death (*p* = 0.225 and *p* = 0.362, respectively), CD (*p* = 0.205 and *p* = 0.216, respectively), NCD (*p* = 0.656 and *p* = 0.956, respectively), recurrent MI (*p* = 0.481 and *p* = 0.520, respectively), any repeat revascularization (*p* = 0.102 and *p* = 0.352, respectively), stroke (*p* = 0.086 and *p* = 0.093, respectively), and ST (*p* = 0.716 and *p* = 0.634, respectively) rates were not significantly different between the SDT < 24 h and SDT ≥ 24 h groups. In the total study population, after multivariable-adjusted and PS-adjusted analyses, all-cause death (*p* = 0.001 and *p* = 0.003, respectively) and CD (*p* = 0.002 and *p* = 0.003, respectively) were significantly higher in the SDT ≥ 24 h group than in the SDT < 24 h group. Table S6 shows the independent predictors of MACCE. Old age (≥65 years, *p* = 0.019 and *p* = 0.012, respectively), reduced LVEF (<50%, *p* = 0.014 and *p* < 0.001, respectively), cardiogenic shock (*p* = 0.041 and *p* = 0.021, respectively), CPR on admission (*p* < 0.001 and *p* < 0.001, respectively), atypical chest pain (*p* < 0.001 and *p* < 0.001, respectively), EMS use (*p* = 0.030 and *p* = 0.039, respectively), and high GRACE risk scores (>140, *p* < 0.001 and *p* < 0.001, respectively) were common independent predictors of MACCE in both the SDT < 24 h and SDT ≥ 24 h groups. As shown in Table S7, old age (*p* < 0.001 and *p* < 0.001, respectively), reduced LVEF (*p* < 0.001 and *p* < 0.001, respectively), CPR on admission (*p* < 0.001 and *p* < 0.001, respectively), atypical chest pain (*p* < 0.001 and *p* < 0.001, respectively), and high GRACE risk scores (*p* = 0.004 and *p* < 0.001, respectively) were common independent predictors of all-cause death in both SDT < 24 h and SDT ≥ 24 h groups. Figures S2 and S3 show the results of subgroup analyses for MACCE and all-cause death in the SDT < 24 h and SDT ≥ 24 h groups using the Cox logistic regression model. In the SDT ≥ 24 h group, all subgroups, except for those showing significant *p*-for-interaction, demonstrated comparable MACCE and all-cause death rates between the male and female groups. In the SDT < 24 h group, however, the female group had a higher all-cause death rate compared with the male group in patients with young age (<65 years, *p* = 0.011) and hypertension (*p* = 0.026). 

## 4. Discussion

The main findings of this prospective observational study after multivariable- and PS-adjusted analyses were as follows: (1) in-hospital mortalities (all-cause death and CD) were not significantly different between the male and female groups in both the SDT < 24 h and SDT ≥ 24 h groups; (2) however, during a 3-year follow-up period in the SDT < 24 h group, all-cause death and CD rates were significantly higher in the female group than those in the male group; (3) furthermore, in the male group, all-cause death and CD rates were significantly lower in the SDT < 24 h group than those in the SDT ≥ 24 h group; (4) MACCE, non-CD, recurrent MI, any repeat revascularization, stroke, and ST rates were similar between the male and female groups and between SDT < 24 h and SDT ≥ 24 h groups; (5) old age, reduced LVEF, CPR on admission, atypical chest pain, and high GRACE risk scores were common independent predictors of MACCE and all-cause death in both the SDT < 24 h and SDT ≥ 24 h groups.

The effects of delayed hospitalization on long-term clinical outcomes in patients with NSTEMI are not well-illuminated, and very limited data are available to date [20,21]. Additionally, the effects of sex differences in delayed hospitalization on long-term clinical outcomes in patients with NSTEMI who were confined to receiving new-generation drug-eluting stent implantation have not been reported. Materic et al. [7] reported that all-cause mortality was higher in females (adjusted odds ratio, 1.03; 95% CI, 1.02–1.04; *p* < 0.001) than in males among 7,026,432 AMI hospitalizations between 2004 and 2015 in the National Inpatient Sample. In our study, in the SDT < 24 h group and total study population, all-cause death and CD were significantly higher in females than in males. In the SDT ≥ 24 h group, these mortalities were also numerically higher in the female group without reaching statistical significance compared with the male group (Table 2). In other words, regarding these results, we may consider that the long-term clinical outcome of the female with NSTEMI after new-generation PCI implantation could be worse than that of the male, regardless of SDT. In particular, the worse long-term clinical outcome was more obvious in the SDT < 24 h group than in the SDT ≥ 24 h group. Additionally, different clinical outcomes according to sex differences in our study were related to relatively low all-cause death and CD rates in the SDT < 24 h group compared to the SDT ≥ 24 h group in male patients (Table 3). It is suggested that females hospitalized with AMI have a higher chance of having worse outcomes than males [8,34]. Females with AMI are older at presentation, have more comorbidity, and present later and with more atypical symptoms [10,11,12,35]. In our study, both in the SDT < 24 h and SDT ≥ 24 h groups, the mean ages of the female group were significantly higher than those in the male group (70.9 ± 9.8 years vs. 61.0 ± 11.6 years; *p* < 0.001, 72.7 ± 9.2 years vs. 63.4 ± 11.9 years; *p* < 0.001, respectively, Table 1). In both the SDT < 24 h and SDT ≥ 24 h groups, the number of patients with hypertension (*p* < 0.001 and *p* < 0.001, respectively), DM (as the abbreviation ”DM” was introduced in the introduction section) (*p* < 0.001 and *p* = 0.005, respectively), high GRACE risk scores (*p* < 0.001 and *p* < 0.001, respectively), and the left anterior descending artery as a treated vessel (*p* = 0.012 and *p* = 0.002, respectively) was also significantly higher in the female group than in the male group (Table 1). Moreover, the number of patients with atypical chest pain was higher in the female group than in the male group (18.1% vs. 12.2%, *p* < 0.001, and 27.9% vs. 20.8%, *p* = 0.006, respectively; Table 1). Old age and atypical chest pain were significant independent predictors of MACCE and all-cause death in both the SDT < 24 h and SDT ≥ 24 h groups (Tables S6 and S7). A recent report [36] (Seems like it could be omitted in the context) showed that females were more likely than males to have atypical symptoms and that women were less likely than males to recognize that their symptoms were due to AMI. Our results showed that all-cause death and CD rates in the female group were higher than those in the male group in the SDT < 24 h group.

In patients with AMI, any delay from symptom onset to treatment is related to a further increase in infarct size and mortality [37] SDT and DBT make up the total ischemic time, and total ischemic time is suggested to be a better predictor of mortality and infarct size than DBT in patients with STEMI [15,38]. Foo et al. [39] showed that the impact of DBT reduction tends to be more significant when the SDT is longer than when it is shorter. In our study, DBT was not an independent predictor of MACCE in both the SDT < 24 h (*p* = 0.311) and SDT ≥ 24 h (*p* = 0.176) groups. Additionally, DBT was not an independent predictor of all-cause death in both the SDT < 24 h (*p* = 0.817) and SDT ≥ 24 h (*p* = 0.203) groups. This result is consistent with the previous results [20,40]. Hence, efforts to reduce delayed hospitalization may be important in reducing mortality in patients with NSTEMI [20]. In our study, although the mean DBT values between the male and female groups in both the SDT < 24 h and SDT ≥ 24 h groups were not significantly different (Table 1), in the male group, the 3-year all-cause death (aHR, 1.450; *p* = 0.010) and CD (aHR, 1.542; *p* = 0.022) rates were higher in the SDT ≥ 24 h group than in the SDT < 24 h group. Similarly, in the total study population, the 3-year all-cause death (aHR, 1.433; *p* = 0.001) and CD (aHR, 1.574; *p* = 0.002) rates were higher in the SDT ≥ 24 h group than in the SDT < 24 h group. However, in the SDT ≥ 24 h group, the 3-year primary and secondary clinical outcomes (Table 2) were not significantly different between the male and female patients. Regarding these results, as mentioned [20,37], SDT could be considered a more important factor than sex difference, even if female patients were older at presentation and had higher comorbidity and atypical chest pain. 

Prehospital delay is the total amount of time taken by patients to present to the emergency department following the onset of acute symptoms [13]. In a recent report [20], patients with NSTEMI and delayed hospitalization had a higher long-term all-cause mortality (17.0% vs. 10.5%; *p* < 0.001) than those without delayed hospitalization. Because they [20] included as many all-comers as possible, these data are valuable in showing the clinical importance of pre-hospital delay in patients with NSTEMI. However, approximately 15% of this study population did not receive PCI or had unsuccessful PCI. Furthermore, patients who received bare-metal stents or first-generation DES were included. To date, second-generation (2G)-DES is the preferred revascularization option because it can reduce restenosis and mortality rates compared with 1G-DES during a long-term follow-up period [41]. In these areas, their research [20] has limitations in terms of reflecting the current real-world practice and demonstrating the long-term prognosis of NSTEMI patients. To overcome these limitations, we excluded patients who did not undergo PCI or who received bare-metal stents or first-generation DES, as shown in Figure 1. In our subgroup analysis, female patients in the SDT < 24 h group who were younger (<65 years) and hypertensive had a higher all-cause death rate than male patients (Figure S2). Champney et al. [42] demonstrated that for both STEMI and NSTEMI, the younger the patient’s age, the higher the mortality risk for women compared to males. Younger patients tend to have more risk factors, such as smoking, obesity, hypertension, dyslipidemia, and a family history of coronary artery disease [43].

In our study, in-hospital mortality, including all-cause death and CD, was not significantly different between the male and female groups in both the SDT < 24 h and SDT ≥ 24 h groups (Table 2). Despite this debate, Heer et al. [44] reported that there were no sex-related differences in in-hospital mortality among 48,215 patients undergoing PCI for NSTEMI between 2007 and the end of 2009. Another registry study [45] also showed that in-hospital mortality did not differ according to sex (adjusted odds ratio [OR], 0.92; 95% CI, 0.57–1.48). 

As far as we know, no specific large-scale study exists, and we could not provide comparative results between our study and other studies. In addition, the population size was insufficient to conclude that the KAMIR-NIH data included 20 tertiary, high-volume university hospitals. Hence, we believe that our results may be the first to compare the long-term clinical outcomes between the SDT < 24 h and SDT ≥ 24 h groups in male and female patients after the successful implantation of new-generation DES and could provide valuable information to cardiologists. 

This study had some limitations. Delayed hospitalization was divided into three phases: patient decision, time to first medical contact, and transportation phases [46]. However, the time variables included in these phases were not included in the KAMIR-NIH data. Hence, information concerning transfers, distance to the nearest hospital, rural vs. urban residence, and the presence or absence of large differences between hospitals in the percentage of patients with delayed hospitalization were not available in the KAMIR-NIH data because these variables were not mandatory in this registry data. Hence, this information was not included in the analysis. This is a major shortcoming of the present study. Second, in this study, we defined delayed hospitalization as SDT ≥ 24 h. However, the clinical outcomes between the male and female groups in the SDT < 24 h and SDT ≥ 24 h groups can be altered according to different definitions of SDT [47]. Third, some subgroups had relatively small sample sizes; hence, their analyses may be underpowered to detect clinically meaningful differences. Fourth, there may have been some under-reported and/or missing data. Fifth, because of the limitations of the medical insurance system in the Republic of Korea, the use of fractional flow reserves to estimate intermediate lesions was low in this study (Table 1). Sixth, the 3-year follow-up period in this study was relatively short for estimating the long-term clinical outcomes. Finally, we believe that both delayed hospitalization and genetic factors [48] may have had negative effects, particularly in the female group. However, in this study, we did not take into account the aspect of genetic factors, so we were unable to fully evaluate their impact. This is other weak point of this study.

## 5. Conclusions

In this nonrandomized, multicenter, prospective cohort study, in-hospital mortalities were similar between the male and female groups. In our study, females with NSTEMI are older at presentation, have more comorbidity, and present later and with more atypical symptoms, and these factors were independent predictors of mortality. This led to lower all-cause death and CD rates (*p* = 0.022 and *p* = 0.012, respectively) in the SDT < 24 h group than in the SDT ≥ 24 h group among male patients. Hence, female patients showed higher 3-year mortality, especially in the SDT < 24 h group and in the total study population, than male patients. However, further large-scale studies are required to confirm our results.

## Figures and Tables

**Table 1:** Baseline characteristics.

Variables	SDT < 24 h, *n* = 3317			SDT ≥ 24 h, *n* = 1276		
	Male (*n* = 2492, Group A)	Female (*n* = 825, Group B)	*p* Value	Male (*n* = 849, Group C)	Female (*n* = 427, Group D)	*p* Value
Age, years	61.0 ± 11.6	70.9 ± 9.8	<0.001	63.4 ± 11.9	72.7 ± 9.2	<0.001
LVEF, %	54.9 ± 10.2	53.7 ± 10.9	0.007	53.6 ± 11.5	53.1 ± 11.5	0.435
BMI, kg/m^2^	24.4 ± 3.2	23.4 ± 3.5	<0.001	24.2 ± 3.3	23.5 ± 3.7	0.003
SBP, mmHg	136.7 ± 26.6	136.7 ± 27.6	0.979	133.4 ± 23.3	133.6 ± 25.6	0.880
DBP, mmHg	82.5 ± 16.0	80.0 ± 15.1	<0.001	80.6 ± 13.9	79.2 ± 15.1	0.116
SDT, hours	3.5 (1.6–8.0)	4.4 (2.0–9.2)	0.001	72.0 (34.7–136.7)	67.9 (32.2–12.0)	0.987
DBT, hours	13.0 (3.9–25.2)	14.1 (4.0–25.0)	0.319	16.5 (3.7–25.6)	16.8 (4.3–26.2)	0.203
Cardiogenic shock, *n* (%)	48 (1.9)	18 (2.2)	0.666	6 (0.7)	9 (2.1)	0.049
CPR on admission, *n* (%)	69 (2.8)	39 (4.7)	0.009	18 (2.1)	16 (3.7)	0.098
Atypical chest pain, *n* (%)	303 (12.2)	149 (18.1)	<0.001	177 (20.8)	119 (27.9)	0.006
Dyspnea, *n* (%)	531 (21.3)	224 (27.2)	0.001	234 (27.6)	140 (32.8)	0.059
EKG on admission						
Q-wave, *n* (%)	190 (7.6)	39 (4.7)	0.004	100 (11.8)	33 (7.7)	0.026
ST-segment depression, *n* (%)	552 (22.2)	227 (27.5)	0.002	135 (15.9)	109 (25.5)	<0.001
T-wave inversion, *n* (%)	468 (18.8)	248 (30.1)	<0.001	211 (24.9)	139 (32.6)	0.004
Atrial fibrillation, *n* (%)	99 (4.0)	36 (4.4)	0.612	31 (3.7)	20 (4.7)	0.368
Killip class 1I/III, *n* (%)	310 (12.4)	177 (21.5)	<0.001	136 (16.0)	105 (24.6)	<0.001
First medical contact						
EMS, *n* (%)	318 (12.8)	104 (12.6)	0.952	30 (3.5)	16 (3.7)	0.874
Non-PCI center, *n* (%)	1207 (48.4)	470 (57.0)	<0.001	484 (57.0)	265 (62.1)	0.092
PCI center, *n* (%)	967 (38.8)	251 (30.4)	<0.001	335 (39.5)	146 (34.2)	0.076
Hypertension, *n* (%)	1163 (46.7)	562 (68.1)	<0.001	423 (49.8)	304 (71.2)	<0.001
Diabetes mellitus, *n* (%)	653 (26.2)	316 (38.3)	<0.001	275 (32.4)	173 (40.5)	0.005
Dyslipidemia, *n* (%)	302 (12.1)	98 (11.9)	0.902	106 (12.5)	42 (9.8)	0.194
Previous MI, *n* (%)	178 (7.1)	49 (5.9)	0.265	61 (7.2)	32 (7.5)	0.821
Previous PCI, *n* (%)	267 (10.7)	78 (9.5)	0.324	80 (9.4)	38 (8.9)	0.838
Previous CABG, *n* (%)	16 (0.6)	9 (1.1)	0.243	7 (0.8)	4 (0.9)	0.838
Previous HF, *n* (%)	29 (1.2)	20 (2.4)	0.012	10 (1.2)	10 (2.3)	0.150
Previous stroke, *n* (%)	128 (5.1)	61 (7.4)	0.019	55 (6.5)	34 (8.0)	0.352
Current smokers, *n* (%)	1201 (48.2)	63 (7.6)	<0.001	358 (42.2)	23 (5.4)	<0.001
Peak CK-MB, mg/dL	28.5 (7.3–94.9)	20.0 (6.6–85.6)	0.034	11.4 (4.3–37.6)	12.8 (5.1–39.9)	0.623
Peak troponin-I, ng/mL	8.6 (1.7–26.9)	6.1 (1.5–22.9)	0.290	4.4 (1.2–16.1)	4.2 (1.2–15.4)	0.264
Blood glucose, mg/dL	156.2 ± 72.3	179.2 ± 93.5	<0.001	146.7 ± 57.1	164.7 ± 97.6	0.001
Hs-CRP (mg/dL)	0.96 ± 2.72	1.60 ± 8.07	0.083	3.02 ± 13.5	2.48 ± 12.8	0.604
Serum creatinine (mg/L)	1.16 ± 1.29	1.08 ± 1.33	0.143	1.25 ± 1.55	1.09 ± 0.98	0.027
Total cholesterol, mg/dL	179.2 ± 43.9	182.4 ± 48.6	0.106	173.2 ± 43.3	180.8 ± 47.8	0.006
Triglyceride, mg/L	136.0 ± 116.9	126.9 ± 130.2	0.088	132.0 ± 110.4	121.0 ± 69.5	0.038
HDL cholesterol, mg/L	42.4 ± 11.3	45.3 ± 12.3	<0.001	40.4 ± 11.2	44.3 ± 12.9	<0.001
LDL cholesterol, mg/L	113.8 ± 38.5	113.8 ± 40.2	0.984	109.3 ± 37.7	114.4 ± 40.6	0.047
GRACE risk score	124.3 ± 40.1	146.7 ± 42.3	<0.001	127.9 ± 36.0	148.4 ± 37.7	<0.001
>140, *n* (%)	683 (27.4)	408 (49.5)	<0.001	273 (32.2)	239 (56.0)	<0.001
Discharge medications, *n* (%)						
Aspirin, *n* (%)	2462 (98.8)	815 (98.8)	0.985	832 (98.0)	423 (99.1)	0.242
Clopidogrel, *n* (%)	1720 (69.0)	643 (77.9)	<0.001	597 (70.3)	362 (84.8)	<0.001
Ticagrelor, *n* (%)	500 (20.1)	144 (17.5)	0.104	160 (18.8)	50 (11.7)	0.001
Prasugrel, *n* (%)	272 (10.9)	38 (4.6)	<0.001	92 (10.8)	15 (3.5)	<0.001
BBs, *n* (%)	2100 (84.3)	714 (86.5)	0.117	711 (83.7)	352 (82.4)	0.578
ACEI or ARBs, *n* (%)	2032 (81.5)	690 (83.6)	0.191	687 (80.9)	344 (80.6)	0.880
Statin, *n* (%)	2362 (94.8)	763 (92.5)	0.016	795 (93.6)	398 (93.2)	0.810
Anticoagulant, *n* (%)	43 (1.7)	14 (1.7)	0.956	23 (2.7)	16 (3.7)	0.306
Infarct-related artery						
Left main, *n* (%)	81 (3.3)	16 (1.9)	0.056	30 (3.5)	19 (4.4)	0.442
LAD, *n* (%)	1045 (41.9)	370 (44.8)	0.143	334 (39.3)	202 (47.3)	0.007
LCx, *n* (%)	666 (26.7)	209 (25.3)	0.439	200 (23.6)	92 (21.5)	0.438
RCA, *n* (%)	700 (28.1)	230 (27.9)	0.929	285 (33.6)	114 (26.7)	0.013
Treated vessel						
Left main, *n* (%)	118 (4.7)	28 (3.4)	0.117	51 (6.0)	26 (6.1)	0.954
LAD, *n* (%)	1385 (55.6)	500 (60.6)	0.012	475 (55.9)	278 (65.1)	0.002
LCx, *n* (%)	976 (39.2)	312 (37.8)	0.510	324 (38.2)	159 (37.2)	0.760
RCA, *n* (%)	914 (36.7)	300 (36.4)	0.900	366 (43.1)	150 (35.1)	0.007
Multivessel disease, *n* (%)	1327 (53.3)	465 (56.4)	0.126	499 (58.8)	250 (58.5)	0.952
ACC/AHA type B2/C lesions, *n* (%)	2100 (84.3)	693 (84.0)	0.869	716 (84.3)	360 (84.3)	0.991
Pre-PCI TIMI flow grade 0/1, *n* (%)	964 (38.7)	315 (38.2)	0.805	338 (39.8)	145 (34.0)	0.044
GP IIb/IIIa inhibitor, *n* (%)	222 (8.9)	66 (8.0)	0.476	77 (9.1)	34 (8.0)	0.530
Transradial approach, *n* (%)	1301 (52.2)	369 (44.7)	<0.001	495 (58.3)	219 (51.3)	0.020
IVUS/OCT, *n* (%)	661 (26.5)	170 (20.6)	0.001	233 (27.4)	87 (20.4)	0.006
FFR, *n* (%)	59 (2.4)	15 (1.8)	0.415	26 (3.1)	3 (0.7)	0.008
Drug-eluting stents ^a^						
ZES, *n* (%)	629 (25.2)	200 (24.2)	0.578	183 (21.6)	98 (23.0)	0.568
EES, *n* (%)	1313 (52.7)	437 (53.0)	0.936	453 (53.4)	230 (53.9)	0.905
BES, *n* (%)	489 (19.6)	166 (20.1)	0.762	198 (23.3)	88 (20.6)	0.287
Others, *n* (%)	61 (2.4)	22 (2.7)	0.701	15 (1.8)	11 (2.6)	0.401
Stent diameter (mm)	3.11 ± 0.43	2.98 ± 0.39	<0.001	3.11 ± 0.43	2.97 ± 0.39	<0.001
Stent length (mm)	29.2 ± 13.7	30.2 ± 14.1	0.078	30.3 ± 15.4	29.9 ± 14.1	0.564
Number of stents	1.19 ± 0.45	1.22 ± 0.46	0.192	1.22 ± 0.47	1.21 ± 0.47	0.863

Values are means ± standard deviation or median (interquartile range) or numbers and percentages. The *p* values for continuous data were obtained from the unpaired *t*-test. The *p* values for categorical data were obtained from the chi-square or Fisher’s exact test. LVEF, left ventricular ejection fraction; BMI, body mass index; SBP, systolic blood pressure; DBP, diastolic blood pressure; SDT, symptom-to-door time; DBT, door-to-balloon time; CPR, cardiopulmonary resuscitation; EKG, electrocardiogram; EMS, emergency medical service; PCI, percutaneous coronary intervention; MI, myocardial infarction; CABG, coronary artery bypass graft; HF, heart failure; CK-MB, creatine kinase myocardial band; Hs-CRP, high sensitivity-c-reactive protein; HDL, high-density lipoprotein; LDL, low-density lipoprotein; GRACE, Global Registry of Acute Coronary Events; BBs, beta-blockers; ACEIs, angiotensin converting enzyme inhibitors; ARBs, angiotensin receptor blockers; LAD, left anterior descending coronary artery; LCx, left circumflex coronary artery; RCA, right coronary artery; ACC/AHA, American College of Cardiology/American Heart Association; TIMI, Thrombolysis In Myocardial Infarction; GP, glycoprotein; IVUS, intravascular ultrasound; OCT, optical coherence tomography; FFR, fractional flow reserve; ZES, zotarolimus-eluting stent; EES, everolimus-eluting stent; BES, biolimus-eluting stent. ^a^ Drug-eluting stents were composed of ZES (Resolute integrity stent; Medtronic, Inc., Minneapolis, MN, USA), EES (Xience Prime stent, Abbott Vascular, Santa Clara, CA, USA; or Promus Element stent, Boston Scientific, Natick, MA, USA), and BES (BioMatrix Flex stent, Biosensors International, Morges, Switzerland; or Nobori stent, Terumo Corporation, Tokyo, Japan).

**Table 2:** Clinical outcomes between male and female groups in patient with or without delayed hospitalization.

	In-Hospital Outcomes								
Outcomes	SDT < 24 h, *n* = 3317								
	Male (*n* = 2492, Group A)	Female (*n* = 825, Group B)	Log-Rank	Unadjusted		Multivariable-Adjusted ^a^		Propensity Score-Adjusted	
				HR (95% CI)	*p*	HR (95% CI)	*p*	HR (95% CI)	*p*
All-cause death	38 (1.5)	16 (1.9)	0.412	0.784 (0.437–1.406)	0.414	1.034 (0.572–1.869)	0.913	1.146 (0.627–2.096)	0.657
Cardiac death	25 (1.0)	13 (1.6)	0.180	0.634 (0.325–1.241)	0.184	1.324 (0.669–2.617)	0.420	1.478 (0.730–2.992)	0.278
	SDT ≥ 24 h, *n* = 1276								
	Male (*n* = 849, group C)	Female (*n* = 427, group D)	Log-rank	Unadjusted		Multivariable-adjusted ^a^		Propensity score-adjusted	
				HR (95% CI)	*p*	HR (95% CI)	*p*	HR (95% CI)	*p*
All-cause death	13 (1.5)	6 (1.4)	0.864	1.088 (0.414–2.864)	0.864	1.218 (0.435–3.411)	0.707	1.029 (0.318–3.218)	0.932
Cardiac death	11 (1.3)	5 (1.2)	0.853	1.105 (0.384–3.180)	0.853	1.011 (0.346–2.952)	0.984	1.087 (0.484–3.380)	0.878
	**3-year outcomes**								
Outcomes	SDT < 24 h, *n* = 3317								
	Male (*n* = 2492, group A)	Female (*n* = 825, group B)	Log-rank	Unadjusted		Multivariable-adjusted ^a^		Propensity score-adjusted	
				HR (95% CI)	*p*	HR (95% CI)	*p*	HR (95% CI)	*p*
MACCE	392 (15.7)	161 (19.5)	0.010	0.783 (0.652–0.941)	0.009	1.181 (0.982–1.422)	0.098	1.208 (1.001–1.462)	0.058
All-cause death	171 (6.9)	88 (10.7)	<0.001	0.631 (0.488–0.816)	<0.001	1.392 (1.073–1.804)	0.013	1.451 (1.118–1.884)	0.005
Cardiac death	97 (3.9)	54 (6.5)	0.001	0.585 (0.419–0.816)	0.002	1.520 (1.086–2.128)	0.015	1.583 (1.129–2.220)	0.008
Non-cardiac death	74 (3.0)	34 (4.2)	0.089	0.705 (0.469–1.058)	0.091	1.226 (0.814–1.845)	0.330	1.282 (0.851–1.933)	0.235
Recurrent MI	73 (3.0)	35 (4.3)	0.050	0.670 (0.448–1.003)	0.052	1.390 (0.926–2.087)	0.112	1.401 (0.932–2.106)	0.105
Any repeat revascularization	224 (9.4)	72 (9.3)	0.998	1.001 (0.767–1.304)	0.999	1.038 (0.794–1.356)	0.785	1.021 (0.781–1.335)	0.879
Stroke	38 (1.6)	20 (2.6)	0.073	0.612 (0.356–1.052)	0.076	1.562 (0.905–2.696)	0.109	1.547 (0.894–2.676)	0.147
ST (definite or probable)	11 (0.5)	6 (0.8)	0.299	0.594 (0.220–1.606)	0.305	1.561 (0.565–4.311)	0.390	1.438 (0.514–4.019)	0.489
Outcomes	SDT ≥ 24 h, *n* = 1276								
	Male (*n* = 849, group C)	Female (*n* = 427, group D)	Log-rank	Unadjusted		Multivariable-adjusted ^a^		Propensity score-adjusted	
				HR (95% CI)	*p*	HR (95% CI)	*p*	HR (95% CI)	*p*
MACCE	146 (17.2)	76 (17.8)	0.835	0.971 (0.736–1.281)	0.835	1.013 (0.766–1.340)	0.927	1.088 (0.819–1.447)	0.561
All-cause death	79 (9.3)	54 (12.7)	0.072	0.729 (0.516–1.031)	0.074	1.375 (0.968–1.952)	0.075	1.362 (0.681–1.842)	0.087
Cardiac death	49 (5.7)	35 (8.2)	0.103	0.699 (0.453–1.078)	0.105	1.484 (0.955–2.305)	0.079	1.451 (0.933–2.214)	0.095
Non-cardiac death	30 (3.6)	19 (4.5)	0.409	0.785 (0.442–1.395)	0.410	1.209 (0.676–2.164)	0.522	1.295 (0.782–2.352)	0.245
Recurrent MI	27 (3.3)	15 (3.7)	0.735	0.897 (0.477–1.686)	0.735	1.099 (0.581–2.078)	0.772	1.143 (0.601–2.175)	0.746
Any repeat revascularization	71 (8.8)	21 (5.2)	0.028	1.715 (1.054–2.790)	0.030	1.534 (0.879–2.676)	0.132	1.597 (0.902–2.987)	0.101
Stroke	22 (2.7)	19 (4.7)	0.072	0.573 (0.310–1.059)	0.076	1.717 (0.923–3.194)	0.088	1.612 (0.884–3.567)	0.198
ST (definite or probable)	6 (0.7)	2 (0.5)	0.620	1.495 (0.302–7.406)	0.623	1.447 (0.286–7.310)	0.655	1.363 (0.189–9.834)	0.758
Outcomes		Total, *n* = 4593							
	Male (*n* = 3341, group A + C)	Female (*n* = 1252, group B + D)	Log-rank	Unadjusted		Multivariable-adjusted ^a^		Propensity score-adjusted	
				HR (95% CI)	*p*	HR (95% CI)	*p*	HR (95% CI)	*p*
MACCE	538 (16.1)	237 (18.9)	0.023	0.837 (0.719–0.976)	0.023	1.124 (0.964–1.311)	0.136	1.131 (1.002–1.383)	0.101
All-cause death	250 (7.5)	142 (11.3)	<0.001	0.649 (0.528–0.797)	<0.001	1.411 (1.147–1.737)	0.001	1.408 (1.131–1.687)	0.002
Cardiac death	146 (4.4)	89 (7.1)	<0.001	0.606 (0.469–0.789)	<0.001	1.525 (1.169–1.989)	0.002	1.458 (1.133–1.563)	0.004
Non-cardiac death	104 (3.1)	53 (4.2)	0.052	0.721 (0.518–1.004)	0.053	1.257 (0.901–1.754)	0.179	1.179 (0.835–1.665)	0.349
Recurrent MI	100 (3.0)	50 (4.1)	0.071	0.732 (0.521–1.028)	0.072	1.300 (0.923–1.830)	0.133	1.286 (0.905–1.785)	0.204
Any repeat revascularization	295 (9.3)	93 (7.9)	0.190	1.168 (0.926–1.475)	0.191	1.230 (0.944–1.603)	0.124	1.181 (0.933–1.594)	0.166
Stroke	60 (1.9)	39 (3.3)	0.005	0.562 (0.376–0.841)	0.005	1.206 (0.753–1.931)	0.437	1.360 (0.814–2.108)	0.183
ST (definite or probable)	17 (0.5)	8 (0.7)	0.564	0.782 (0.337–1.811)	0.565	1.266 (0.542–2.957)	0.585	1.301 (0.787–3.124)	0.202

SDT, symptom-to-door time; HR hazard ratio, CI confidence interval, MACCE, major adverse cardiac and cerebrovascular events; MI, myocardial infarction; ST stent thrombosis, LVEF, left ventricular ejection fraction; BMI, body mass index; DBP, diastolic blood pressure; DBT, door-to-balloon time; CPR, cardiopulmonary resuscitation; PCI, percutaneous coronary intervention; CK-MB, creatine kinase myocardial band; HDL, high-density lipoprotein; LDL, low-density lipoprotein. ^a^ Adjusted by age, LVEF, BMI, DBP, DBT, cardiogenic shock, CPR on admission, atypical chest pain, dyspnea, Q-wave, ST-segment depression, and T wave inversion on EKG, Killip class II/III, non-PCI center, PCI center, hypertension, diabetes mellitus, previous heart failure, previous stroke, current smoker, peak CK-MB, blood glucose, serum creatinine, total cholesterol, triglyceride, HDL-cholesterol, and LDL-cholesterol (Table S1).

**Table 3:** Clinical outcomes between the SDT < 24 h and SDT ≥ 24 h groups according to sex difference.

	In-Hospital Outcomes								
Outcomes	Male, *n* = 3341								
	SDT < 24 h (*n* = 2492, Group A)	SDT ≥ 24 h (*n* = 849, Group C)	Log-Rank	Unadjusted		Multivariable-Adjusted ^a^		Propensity Score-Adjusted	
				HR (95% CI)	*p*	HR (95% CI)	*p*	HR (95% CI)	*p*
All-cause death	38 (1.5)	13 (1.5)	0.985	0.994 (0.530–1.866)	0.985	1.425 (0.705–2.883)	0.327	1.468 (0.750–3.012)	0.238
Cardiac death	25 (1.0)	11 (1.3)	0.477	0.774 (0.381–1.572)	0.478	2.036 (0.871–4.758)	0.101	2.141 (0.917–4.784)	0.096
Outcomes	Female, *n* = 1252								
	SDT < 24 h (*n* = 825, group B)	SDT ≥ 24 h (*n* = 427, group D)	Log-rank	Unadjusted		Multivariable-adjusted ^a^		Propensity score-adjusted	
				HR (95% CI)	*p*	HR (95% CI)	*p*	HR (95% CI)	*p*
All-cause death	16 (1.9)	6 (1.4)	0.498	1.381 (0.540–3.530)	0.500	1.143 (0.377–3.463)	0.813	1.198 (0.443–3.621)	0.622
Cardiac death	13 (1.6)	5 (1.2)	0.570	1.346 (0.480–3.777)	0.572	1.618 (0.488–5.363)	0.432	1.697 (0.561–6.021)	0.387
	**3-year outcomes**								
Outcomes	Male, *n* = 3341								
	SDT < 24 h (*n* = 2492, group A)	SDT ≥ 24 h (*n* = 849, group C)	Log-rank	Unadjusted		Multivariable-adjusted ^a^		Propensity score-adjusted	
				HR (95% CI)	*p*	HR (95% CI)	*p*	HR (95% CI)	*p*
MACCE	392 (15.7)	146 (17.2)	0.331	0.910 (0.753–1.100)	0.331	1.099 (0.901–1.341)	0.351	1.110 (0.912–1.349)	0.297
All-cause death	171 (6.9)	79 (9.3)	0.020	0.730 (0.559–0.953)	0.020	1.450 (1.066–1.925)	0.010	1.434 (1.085–1.894)	0.011
Cardiac death	97 (3.9)	49 (5.7)	0.021	0.668 (0.474–0.942)	0.021	1.542 (1.093–2.231)	0.022	1.587 (1.105–2.280)	0.012
Non-cardiac death	74 (3.0)	30 (3.6)	0.390	0.830 (0.543–1.269)	0.390	1.291 (0.827–2.016)	0.261	1.251 (0.806–1.943)	0.318
Recurrent MI	73 (3.0)	27 (3.3)	0.673	0.909 (0.585–1.414)	0.673	1.105 (0.698–1.748)	0.670	1.033 (0.658–1.622)	0.887
Any repeat revascularization	224 (9.4)	71 (8.8)	0.636	1.066 (0.817–1.393)	0.637	1.132 (0.860–1.491)	0.377	1.095 (0.834–1.438)	0.515
Stroke	38 (1.6)	22 (2.7)	0.037	0.570 (0.341–0.975)	0.040	1.378 (0.806–2.390)	0.238	1.474 (0.860–2.526)	0.158
ST (definite or probable)	11 (0.5)	6 (0.7)	0.336	0.616 (0.228–1.667)	0.340	1.253 (0.443–3.545)	0.670	1.293 (0.466–3.687)	0.612
Outcomes	Female, *n* = 1252								
	SDT < 24 h (*n* = 825, group B)	SDT ≥ 24 h (*n* = 427, group D)	Log-rank	Unadjusted		Multivariable-adjusted ^a^		Propensity score-adjusted	
				HR (95% CI)	*p*	HR (95% CI)	*p*	HR (95% CI)	*p*
MACCE	161 (19.5)	76 (17.8)	0.399	1.124 (0.856–1.477)	0.399	1.107 (0.833–1.471)	0.483	1.133 (0.855–1.512)	0.385
All-cause death	88 (10.7)	54 (12.7)	0.328	0.845 (0.602–1.185)	0.329	1.249 (0.852–1.787)	0.225	1.177 (0.829–1.673)	0.362
Cardiac death	54 (6.5)	35 (8.2)	0.303	0.800 (0.523–1.224)	0.304	1.342 (0.872–2.112)	0.205	1.325 (0.848–2.070)	0.216
Non-cardiac death	34 (4.2)	19 (4.5)	0.790	0.926 (0.528–1.624)	0.790	1.143 (0.635–2.055)	0.656	1.016 (0.573–1.801)	0.956
Recurrent MI	35 (4.3)	15 (3.7)	0.518	1.220 (0.666–2.234)	0.519	1.253 (0.669–2.346)	0.481	1.226 (0.659–2.282)	0.520
Any repeat revascularization	72 (9.3)	21 (5.2)	0.014	1.825 (1.122–2.967)	0.015	1.634 (0.994–2.542)	0.102	1.531 (0.864–2.375)	0.352
Stroke	20 (2.6)	19 (4.7)	0.051	0.541 (0.289–1.013)	0.055	1.706 (0.103–3.376)	0.086	1.698 (0.913–3.264)	0.093
ST (definite or probable)	6 (0.8)	2 (0.5)	0.586	1.555 (0.314–7.703)	0.589	1.395 (0.232–8.387)	0.716	1.451 (0.264–8.874)	0.634
Outcomes		Total, *n* = 4593							
	SDT < 24 h (*n* = 3317, group A + B)	SDT ≥ 24 h (*n* = 1276, group C + D)	Log-rank	Unadjusted		Multivariable-adjusted ^a^		Propensity score-adjusted	
				HR (95% CI)	*p*	HR (95% CI)	*p*	HR (95% CI)	*p*
MACCE	553 (16.7)	222 (17.4)	0.615	0.961 (0.822–1.123)	0.615	1.051 (0.895–1.235)	0.543	1.044 (0.890–1.189)	0.625
All-cause death	259 (7.8)	133 (10.4)	0.004	0.743 (0.603–0.916)	0.005	1.433 (1.152–1.781)	0.001	1.385 (1.115–1.718)	0.003
Cardiac death	151(4.5)	84 (6.5)	0.005	0.687 (0.526–0.896)	0.006	1.574 (1.189–2.082)	0.002	1.543 (1.168–2.039)	0.003
Non-cardiac death	108 (3.3)	49 (3.9)	0.309	0.839 (0.599–1.176)	0.309	1.234 (0.871–1.751)	0.237	1.178 (0.832–1.696)	0.355
Recurrent MI	108 (3.3)	42 (3.5)	0.871	0.981 (0.687–1.401)	0.872	1.134 (0.784–1.639)	0.505	1.082 (0.752–1.558)	0.671
Any repeat revascularization	296 (9.4)	92 (7.6)	0.071	1.240 (0.981–1.567)	0.071	1.231 (0.961–1.418)	0.082	1.214 (0.958–1.540)	0.109
Stroke	58 (1.8)	41 (3.4)	0.002	0.534 (0.358–0.797)	0.002	1.502 (1.062–2.335)	0.182	1.513 (1.074–2.412)	0.142
ST (definite or probable)	17 (0.5)	8 (0.7)	0.620	0.809 (0.349–1.874)	0.620	1.059 (0.438–2.565)	0.898	1.078 (0.450–2.583)	0.832

SDT, symptom-to-door time; HR hazard ratio, CI confidence interval, MACCE, major adverse cardiac and cerebrovascular events; MI, myocardial infarction; ST stent thrombosis, LVEF, left ventricular ejection fraction; BMI, body mass index, SBP, systolic blood pressure; DBP, diastolic blood pressure; DBT, door-to-balloon time; CPR, cardiopulmonary resuscitation; EKG, electrocardiogram; EMS, emergency medical service; PCI, percutaneous coronary intervention; CK-MB, creatine kinase myocardial band; Hs-CRP, high sensitivity-c-reactive protein; HDL, high-density lipoprotein; LDL, low-density lipoprotein. ^a^ Adjusted by age, LVEF, BMI, SBP, DBP, DBT, cardiogenic shock, CPR on admission, atypical chest pain, dyspnea, Q-wave, ST-segment depression, T wave inversion on EKG, Killip class II/III, EMS, non-PCI center, diabetes mellitus, current smoker, peak CK-MB, peak troponin-I, blood glucose, Hs-CRP, serum creatinine, total cholesterol, HDL-cholesterol, and LDL-cholesterol, (Table S3).

## Data Availability

Data is contained within the article or supplementary material.

## Supplementary Materials

The following supporting information can be downloaded at: https://www.mdpi.com/article/10.3390/jcm12051982/s1, Table S1: Results of collinearity test for MACCE between the male and female groups, Table S2: Baseline characteristics between the male and female groups before and after PSM, Table S3: Results of collinearity test for MACCE between the SDT < 24 h and SDT ≥ 24 h groups, Table S4: Baseline characteristics between the male and female groups according to SDT, Table S5: Baseline characteristics between the SDT < 24 h and SDT ≥ 24 h groups before and after PSM, Table S6: Independent predictors for MACCE, Table S7: Independent predictors for all-cause death, Figure S1: Causes of acute myocardial infarction. Figure S2: Subgroup analysis for MACCE in the SDT < 24 h and SDT ≥ 24 h groups, Figure S3: Subgroup analysis for all-cause death in the SDT < 24 h and SDT ≥ 24 h groups.
