# Supplementary material for: Sex Differences in Delayed Hospitalization in Patients with Non-ST-Segment Elevation Myocardial Infarction Undergoing New-Generation Drug-Eluting Stent Implantation
Source: J Clin Med. 2023 Mar 2;12(5):1982. doi: 10.3390/jcm12051982 (PMC10003952; doi:10.3390/jcm12051982)
Supplement: Supplementary file 1 [file jcm-12-01982-s001.zip › jcm-2221891-supplementary.pdf]

Supplementary Materials

Sex differences in delayed hospitalization in patients with non-ST-segment elevation myocardial infarction undergoing new-generation drug-eluting stent implantation

Yong Hoon Kim [1,*,†], Ae-Young Her [1,†], Seung-Woon Rha [2,*], Cheol Ung Choi [2], Byoung Geol Choi [3], Ji Bak Kim [2], Soohyung Park [2], Dong Oh Kang [2], Ji Young Park [4], Woong Gil Choi [5], Sang-Ho Park [6] and Myung Ho Jeong [7]

Supplementary Online Contents

Table S1. Results of collinearity test for MACCE between the male and female groups

Table S2. Baseline characteristics between the male and female groups before and after PSM

Table S3 Results of collinearity test for MACCE between the SDT <24 h and SDT ≥24 h groups

Table S4. Baseline characteristics between the male and female groups according to SDT

Table S5 Baseline characteristics between the SDT <24 h and SDT ≥24 h groups before and after PSM

Table S6. Independent predictors for MACCE

Table S7. Independent predictors for all-cause death

Figure S1. Causes of acute myocardial infarction

Figure S2. Subgroup analysis for MACCE in the SDT <24 h and SDT ≥24 h groups

Figure S3. Subgroup analysis for all-cause death in the SDT <24 h and SDT ≥24 h groups

Table S1. Results of collinearity test for MACCE between the male and female groups.

	Variance Inflation Factors	Tolerance	Condition Index
Age	2.713	0.369	1.000
LVEF	1.264	0.791	3.364
BMI	1.198	0.835	3.752
DBP	1.304	0.767	4.165
DBT	1.066	0.938	4.297
Cardiogenic shock	1.235	0.810	4.386
CPR on admission	1.411	0.709	4.441
Atypical chest pain	1.210	0.826	4.497
Dyspnea	1.215	0.823	4.558
Q-wave	1.054	0.949	4.659
ST-segment depression	1.603	0.624	4.674
T-wave inversion	1.158	0.864	4.736
Killip class II/III	1.630	0.613	4.903
Non-PCI center	3.005	0.333	5.070
PCI center	2.991	0.334	5.094
Hypertension	1.198	0.835	5.156
Diabetes mellitus	1.430	0.699	5.474
Previous HF	1.036	0.965	5.516
Previous stroke	1.045	0.957	5.643
Current smoker	1.258	0.795	5.787
Peak CK-MB	1.091	0.917	6.205
Blood glucose	1.381	0.724	6.244
Serum creatinine	1.166	0.857	6.352
Total cholesterol	3.725	0.268	6.490
Triglyceride	1.294	0.773	7.223
HDL-cholesterol	1.259	0.794	7.403
LDL-cholesterol	3.206	0.312	7.660
GRACE risk score	4.744	0.211	10.902
Clopidogrel	17.070	0.059	12.308
Ticagrelor	13.374	0.075	13.322
Prasugrel	7.693	0.130	14.966
Statin	1.146	0.873	15.574
LAD (IRA)	4.121	0.243	19.091
RCA (IRA)	2.781	0.360	19.147
LAD (treated vessel)	2.268	0.441	20.651
RCA (treated vessel)	2.414	0.414	26.494
Pre-PCI TIMI flow grade 0/1	1.133	0.883	29.685
Transradial approach	1.138	0.879	37.346
IVUS/OCT	1.070	0.935	38.859
FFR	1.014	0.987	42.849
Stent diameter	1.060	0.862	48.074

MACCE, major adverse cardiac and cerebrovascular events; LVEF, left ventricular ejection fraction; BMI, body mass index; DBP, diastolic blood pressure; DBT, door-to-balloon time; CPR, cardiopulmonary resuscitation; EKG, electrocardiogram; PCI, percutaneous coronary intervention; HF, heart failure; CK-MB, creatine kinase myocardial band; HDL, high-density

lipoprotein; LDL. Low-density lipoprotein; GRACE, Global Registry of Acute Coronary Events; LAD, left anterior descending coronary artery; IRA, infarct-related artery; RCA, right coronary artery; TIMI, thrombolysis in myocardial infarction; IVUS, intravascular ultrasound; OCT, optical coherence tomography; FFR, fractional flow reserve

Table S2. Baseline characteristics between the male and female groups before and after PSM.

Variables	Entire patients, n = 4593			PSM patients, n = 1884			
	Male (n = 3341, group A)	Female (n = 1252, group B)	p value	Male (n = 942, group C)	Female (n = 942, group D)	p value	SD
Age, years	61.6 ± 11.7	71.6 ± 9.6	<0.001	69.3 ± 10.1	69.8 ± 9.8	0.334	-0.50
LVEF, %	54.6 ± 10.6	53.5 ± 11.1	0.004	53.3 ± 10.9	53.5 ± 10.9	0.636	-0.18
BMI, kg/m^2	24.43 ± 3.2	23.5 ± 3.6	<0.001	23.6 ± 3.1	23.6 ± 3.5	0.939	0.03
SBP, mmHg	135.9 ± 25.9	135.7 ± 27.0	0.829	135.6 ± 26.4	136.0 ± 26.8	0.763	-0.15
DBP, mmHg	82.0 ± 15.5	79.8 ± 15.1	<0.001	80.0 ± 14.7	80.4 ± 15.2	0.644	-0.27
DBT, hours	13.5 (3.8-25.3)	14.8 (4.2-25.6)	0.060	14.7 (3.9-27.8)	15.4 (4.2-25.5)	0.700	-0.18
Cardiogenic shock, n (%)	54 (1.6)	27 (2.2)	0.134	13 (1.4)	20 (2.1)	0.292	-0.50
CPR on admission, n (%)	87 (2.6)	55 (4.4)	0.003	36 (3.8)	37 (3.9)	0.905	-0.05
Atypical chest pain, n (%)	480 (14.4)	268 (21.4)	<0.001	1857 (19.6)	188 (20.0)	0.908	-0.01
Dyspnea, n (%)	765 (22.9)	364 (29.1)	<0.001	269 (28.6)	262 (27.8)	0.759	0.18
EKG on admission							
Q-wave, n (%)	290 (8.7)	72 (5.8)	0.001	61 (6.5)	66 (7.0)	0.713	-0.19
ST-segment depression, n (%)	687 (20.6)	336 (26.8)	<0.001	231 (24.5)	236 (25.1)	0.831	-0.14
T-wave inversion, n (%)	679 (20.3)	387 (30.9)	<0.001	262 (27.8)	251 (26.6)	0.605	0.27
Atrial fibrillation, n (%)	130 (3.9)	56 (4.5)	0.400	45 (4.8)	46 (4.9)	0.914	-0.05
Killip class 1I/III, n (%)	446 (13.3)	282 (22.5)	<0.001	205 (21.8)	193 (20.5)	0.535	0.32
First medical contact							
EMS, n (%)	348 (10.4)	120 (9.6)	0.443	85 (9.0)	94 (10.0)	0.530	-0.34
Non-PCI center, n (%)	1691 (50.6)	735 (58.7)	<0.001	527 (55.9)	528 (56.1)	0.963	-0.04
PCI center, n (%)	1302 (39.0)	397 (31.7)	<0.001	330 (35.0)	320 (34.0)	0.663	0.21
Hypertension, n (%)	1586 (47.5)	866 (69.2)	<0.001	597 (63.4)	612 (65.0)	0.501	-0.33
Diabetes mellitus, n (%)	928 (27.8)	489 (39.1)	<0.001	355 (37.3)	348 (36.9)	0.775	0.10
Dyslipidemia, n (%)	408 (12.2)	140 (11.2)	0.358	99 (10.5)	106 (11.3)	0.657	-0.25
Previous MI, n (%)	239 (7.2)	81 (6.5)	0.435	71 (7.5)	67 (7.1)	0.791	0.15
Previous PCI, n (%)	347 (10.4)	116 (9.3)	0.271	100 (10.6)	101 (10.7)	0.941	-0.03

Previous CABG, *n* (%)	23 (0.7)	13 (1.0)	0.259	15 (1.6)	11 (1.2)	0.554	0.34
Previous HF, *n* (%)	39 (1.2)	30 (2.4)	0.004	20 (2.1)	21 (2.2)	0.875	-0.07
Previous stroke, *n* (%)	183 (5.5)	95 (7.6)	0.010	78 (8.3)	73 (7.7)	0.734	0.22
Current smokers, *n* (%)	1559 (46.7)	86 (6.9)	<0.001	94 (10.0)	85 (9.0)	0.530	0.34
Peak CK-MB, mg/dL	21.7 (5.9-80.2)	16.6 (5.8-65.9)	0.008	21.0 (6.1-76.1)	17.7 (6.0-66.1)	0.477	0.33
Peak troponin-I, ng/mL	6.9 (1.6-24.8)	5.4 (1.4-19.8)	0.078	7.5 (1.6-23.0)	6.6 (1.6-22.9)	0.406	0.38
Blood glucose, mg/dL	153.9 ± 71.1	174.3 ± 95.1	<0.001	169.8 ± 84.9	169.7 ± 89.8	0.972	0.01
Hs-CRP (mg/dL)	1.45 ± 2.85	1.89 ± 3.05	0.272	1.55 ± 2.86	1.51 ± 2.58	0.776	0.15
Serum creatinine (mg/L)	1.18 ± 1.36	1.08 ± 1.22	0.020	1.16 ± 0.83	1.12 ± 1.37	0.385	0.35
Total cholesterol, mg/dL	177.7 ± 43.8	181.8 ± 48.3	0.009	176.8 ± 43.2	175.3 ± 45.9	0.466	0.34
Triglyceride, mg/L	135.0 ± 115.3	124.9 ± 113.6	0.011	121.0 ± 102.7	118.4 ± 76.3	0.538	0.29
HDL cholesterol, mg/L	41.9 ± 11.3	45.0 ± 12.5	<0.001	44.1 ± 11.7	43.9 ± 11.6	0.606	0.17
LDL cholesterol, mg/L	112.7 ± 38.3	114.0 ± 40.3	0.342	110.3 ± 35.5	110.4 ± 36.8	0.989	-0.02
GRACE risk score	125.2 ± 39.1	147.3 ± 40.8	<0.001	142.9 ± 40.3	143.0 ± 40.5	0.973	-0.02
>140, *n* (%)	956 (28.6)	647 (51.7)	<0.001	442 (46.9)	443 (47.0)	0.963	-0.02
Discharge medications, *n* (%)							
Aspirin, *n* (%)	3294 (98.6)	1238 (98.9)	0.563	929 (98.6)	930 (98.7)	0.840	-0.08
Clopidogrel, *n* (%)	2317 (69.4)	1005 (80.3)	<0.001	719 (76.3)	730 (77.5)	0.585	-0.29
Ticagrelor, *n* (%)	660 (19.8)	194 (15.5)	0.001	160 (17.0)	149 (15.8)	0.534	0.32
Prasugrel, *n* (%)	364 (10.9)	53 (4.2)	<0.001	50 (5.3)	51 (5.4)	0.919	-0.04
BBs, *n* (%)	2811 (84.1)	1066 (85.1)	0.411	798 (84.7)	790 (83.9)	0.658	0.22
ACEI or ARBs, *n* (%)	2719 (81.4)	1034 (82.6)	0.368	753 (79.9)	772 (82.0)	0.291	-0.54
Statin, n (%)	3157 (94.5)	1161 (92.7)	0.030	866 (91.9)	864 (91.7)	0.933	0.07
Anticoagulant, n (%)	66 (2.0)	30 (2.4)	0.417	21 (2.2)	24 (2.5)	0.763	-0.19
Infarct-related artery							
Left main, *n* (%)	111 (3.3)	35 (2.8)	0.396	35 (3.7)	31 (3.3)	0.707	0.22
LAD, *n* (%)	1379 (41.3)	572 (45.7)	0.006	417 (44.3)	418 (44.4)	0.963	-0.02
LCx, *n* (%)	866 (25.9)	301 (24.0)	0.196	216 (22.9)	231 (24.5)	0.448	-0.38
RCA, *n* (%)	985 (29.5)	344 (27.5)	0.188	270 (28.7)	262 (27.8)	0.720	0.20
Treated vessel							

Left main, *n* (%)	169 (5.1)	54 (4.3)	0.317	45 (4.8)	47 (5.0)	0.915	-0.09
LAD, *n* (%)	1860 (55.7)	778 (62.1)	<0.001	563 (59.8)	574 (60.9)	0.638	-0.23
LCx, *n* (%)	1300 (38.9)	471 (37.6)	0.434	335 (35.6)	362 (38.4)	0.215	-0.58
RCA, *n* (%)	1280 (38.3)	450 (35.9)	0.142	362 (38.4)	348 (36.9)	0.537	0.31
Multivessel disease, *n* (%)	1826 (54.7)	715 (57.1)	0.143	547 (58.1)	542 (57.5)	0.852	0.12
ACC/AHA type B2/C lesions, *n* (%)	2816 (84.3)	1053 (84.1)	0.892	790 (83.9)	791 (84.0)	0.950	-0.03
Pre-PCI TIMI flow grade 0/1, *n* (%)	1302 (39.0)	460 (36.7)	0.173	361 (38.3)	339 (36.0)	0.317	0.48
GP IIb/IIIa inhibitor, *n* (%)	299 (8.9)	100 (8.0)	0.318	76 (8.1)	78 (8.3)	0.933	-0.07
Transradial approach, *n* (%)	1796 (53.8)	588 (47.0)	<0.001	437 (46.4)	445 (47.2)	0.747	-0.16
IVUS/OCT, *n* (%)	894 (26.8)	257 (20.5)	<0.001	203 (21.5)	214 (22.7)	0.579	-0.29
FFR, *n* (%)	85 (2.5)	18 (1.4)	0.025	17 (1.8)	16 (1.7)	0.861	0.08
Drug-eluting stents[a]							
ZES, *n* (%)	812 (24.3)	298 (23.8)	0.757	234 (24.8)	228 (24.2)	0.789	0.14
EES, *n* (%)	1766 (52.9)	667 (53.3)	0.815	502 (53.3)	493 (52.3)	0.678	0.20
BES, *n* (%)	687 (20.6)	254 (20.3)	0.870	184 (19.5)	196 (20.8)	0.528	-0.32
Others, *n* (%)	76 (2.3)	33 (2.6)	0.514	22 (2.3)	25 (2.7)	0.768	-0.26
Stent diameter (mm)	3.11 ± 0.43	2.98 ± 0.39	<0.001	3.02 ± 0.39	3.01 ± 0.40	0.619	0.25
Stent length (mm)	29.5 ± 14.1	30.1 ± 14.1	0.222	30.2 ± 14.7	30.0 ± 14.0	0.684	0.14
Number of stents	1.20 ± 0.45	1.22 ± 0.46	0.285	1.21 ± 0.48	1.21 ± 0.46	0.882	0.05

Values are means ± standard deviation or median (interquartile range) or numbers and percentages. The *p* values for continuous data were obtained from the unpaired t-test. The *p* values for categorical data were obtained from the chi-square or Fisher's exact test. LVEF, left ventricular ejection fraction; BMI, body mass index; SBP, systolic blood pressure; DBP, diastolic blood pressure; SDT, symptom-to-door time; DBT, door-to-balloon time; CPR, cardiopulmonary resuscitation; EKG, electrocardiogram; EMS, emergency medical service; PCI, percutaneous coronary intervention; MI, myocardial infarction; CABG, coronary artery bypass graft; HF, heart failure; CK-MB, creatine kinase myocardial band; Hs-CRP, high sensitivity-c-reactive protein; HDL, high-density lipoprotein; LDL, low-density lipoprotein; GRACE, Global Registry of Acute Coronary Events; BBs, beta-blockers; ACEIs, angiotensin converting enzyme inhibitors; ARBs, angiotensin receptor blockers; LAD, left anterior descending coronary artery; LCx, left circumflex coronary artery; RCA, right coronary artery; ACC/AHA, American College of Cardiology/American Heart Association; TIMI, Thrombolysis In Myocardial Infarction; GP, glycoprotein; IVUS, intravascular ultrasound; OCT, optical coherence tomography; FFR, fractional flow reserve; ZES, zotarolimus-eluting stent; EES, everolimus-eluting stent; BES, biolimus-eluting stent. [a] Drug-eluting stents were composed of ZES (Resolute integrity stent; Medtronic, Inc., Minneapolis, MN), EES (Xience Prime stent, Abbott Vascular, Santa Clara, CA; or Promus Element stent, Boston Scientific, Natick, MA), and BES (BioMatrix Flex stent, Biosensors International, Morges, Switzerland; or Nobori stent, Terumo Corporation, Tokyo, Japan).

Table S3. Results of collinearity test for MACCE between the <24 h and ≥24 h groups.

	Variance Inflation Factors	Tolerance	Condition Index
Age	2.848	0.351	1.000
LVEF	1.274	0.785	3.020
BMI	1.167	0.857	3.312
SBP	3.077	0.325	3.701
DBP	2.794	0.358	3.861
DBT	1.077	0.929	4.033
Cardiogenic shock	1.229	0.813	4.097
CPR on admission	1.390	0.719	4.217
Atypical chest pain	1.214	0.824	4.235
Dyspnea	1.221	0.819	4.291
Q-wave in EKG	1.051	0.951	4.415
ST-segment depression	1.661	0.602	4.441
T-wave inversion	1.150	0.869	4.557
Killip class II/III	1.648	0.607	4.833
EMS	1.179	0.848	4.890
Non-PCI center	1.198	0.835	5.032
Diabetes mellitus	1.392	0.719	5.188
Current smoker	1.240	0.806	5.307
Peak CK-MB	1.153	0.867	5.370
Peak troponin-I	1.168	0.856	5.666
Blood glucose	1.385	0.722	5.719
Hs-CRP	1.041	0.961	5.863
Serum creatinine	1.166	0.858	5.914
Total cholesterol	3.233	0.309	6.107
HDL-cholesterol	1.172	0.853	6.726
LDL-cholesterol	2.997	0.334	6.967
GRACE risk score	5.284	0.189	7.331
Clopidogrel	2.426	0.412	9.701
Ticagrelor	2.357	0.424	11.635
Anticoagulant	1.042	0.959	12.264
LMCA (IRA)	2.878	0.348	12.789
RCA (IRA)	3.412	0.293	14.630
LMCA (treated vessel)	2.892	0.346	16.415
RCA (treated vessel)	3.331	0.300	18.048
Multivessel disease	1.243	0.805	29.368
Transradial approach	1.121	0.892	37.799
IVUS/OCT	1.068	0.936	42.989
ZES	1.107	0.903	53.874
BES	1.123	0.890	74.572

MACCE, major adverse cardiac and cerebrovascular events; LVEF, left ventricular ejection fraction; BMI, body mass index; SBP, systolic blood pressure; DBP, diastolic blood pressure; DBT, door-to-balloon time; CPR, cardiopulmonary resuscitation; EKG, electrocardiogram; EMS, emergency medical service; PCI, percutaneous coronary intervention; CK-MB, creatine kinase myocardial band; Hs-CRP, high sensitivity-c-reactive protein; HDL, high-

density lipoprotein; LDL. Low-density lipoprotein; GRACE, Global Registry of Acute Coronary Events; LMCA, left main coronary artery; IRA, infarct-related artery; RCA, right coronary artery; IVUS, intravascular ultrasound; OCT, optical coherence tomography; ZES, zotarolimus-eluting stent; BES, biolimus-eluting stent.

Table S4. Baseline characteristics between the male and female groups according to SDT.

Variables	Male, *n* = 3341			Female, *n* = 1252		
	SDT <24 h (*n* = 2492, group A)	SDT ≥24 h (*n* = 849, group C)	*p* value	SDT <24 h (*n* = 825, group B)	SDT ≥24 h (*n* = 427, group D)	*p* value
Age, years	61.0 ± 11.6	63.4 ± 11.9	<0.001	70.9 ± 9.8	72.7 ± 9.2	0.001
LVEF, %	54.9 ± 10.2	53.6 ± 11.5	0.005	53.7 ± 10.9	53.1 ± 11.5	0.358
BMI, kg/m^2	24.4 ± 3.2	24.2 ± 3.3	0.197	23.4 ± 3.5	23.5 ± 3.7	0.630
SBP, mmHg	136.7 ± 26.6	133.4 ± 23.3	0.001	136.7 ± 27.6	133.6 ± 25.6	0.051
DBP, mmHg	82.5 ± 16.0	80.6 ± 13.9	0.001	80.0 ± 15.1	79.2 ± 15.1	0.388
DBT, hours	13.0 (3.9-25.2)	16.5 (3.7-25.6)	0.032	14.1 (4.0-25.0)	16.8 (4.3-26.2)	0.037
Cardiogenic shock, *n* (%)	48 (1.9)	6 (0.7)	0.017	18 (2.2)	9 (2.1)	0.932
CPR on admission, *n* (%)	69 (2.8)	18 (2.1)	0.382	39 (4.7)	16 (3.7)	0.603
Atypical chest pain, *n* (%)	303 (12.2)	177 (20.8)	<0.001	149 (18.1)	119 (27.9)	<0.001
Dyspnea, *n* (%)	531 (21.3)	234 (27.6)	<0.001	224 (27.2)	140 (32.8)	0.042
EKG on admission						
Q-wave, *n* (%)	190 (7.6)	100 (11.8)	<0.001	39 (4.7)	33 (7.7)	0.040
ST-segment depression, *n* (%)	552 (22.2)	135 (15.9)	<0.001	227 (27.5)	109 (25.5)	0.460
T-wave inversion, *n* (%)	468 (18.8)	211 (24.9)	<0.001	248 (30.1)	139 (32.6)	0.367
Atrial fibrillation, *n* (%)	99 (4.0)	31 (3.7)	0.758	36 (4.4)	20 (4.7)	0.775
Killip class 1I/III, *n* (%)	310 (12.4)	136 (16.0)	0.010	177 (21.5)	105 (24.6)	0.225
First medical contact						
EMS, *n* (%)	318 (12.8)	30 (3.5)	<0.001	104 (12.6)	16 (3.7)	<0.001
Non-PCI center, *n* (%)	1207 (48.4)	484 (57.0)	<0.001	470 (57.0)	265 (62.1)	0.090
PCI center, *n* (%)	967 (38.8)	335 (39.5)	0.745	251 (30.4)	146 (34.2)	0.179
Hypertension, *n* (%)	1163 (46.7)	423 (49.8)	0.121	562 (68.1)	304 (71.2)	0.273
Diabetes mellitus, *n* (%)	653 (26.2)	275 (32.4)	0.001	316 (38.3)	173 (40.5)	0.464
Dyslipidemia, *n* (%)	302 (12.1)	106 (12.5)	0.762	98 (11.9)	42 (9.8)	0.299
Previous MI, *n* (%)	178 (7.1)	61 (7.2)	0.967	49 (5.9)	32 (7.5)	0.332
Previous PCI, *n* (%)	267 (10.7)	80 (9.4)	0.298	78 (9.5)	38 (8.9)	0.837

Previous CABG, *n* (%)	16 (0.6)	7 (0.8)	0.631	9 (1.1)	4 (0.9)	0.799
Previous HF, *n* (%)	29 (1.2)	10 (1.2)	0.974	20 (2.4)	10 (2.3)	0.928
Previous stroke, *n* (%)	128 (5.1)	55 (6.5)	0.138	61 (7.4)	34 (8.0)	0.736
Current smokers, *n* (%)	1201 (48.2)	358 (42.2)	0.002	63 (7.6)	23 (5.4)	0.157
Peak CK-MB, mg/dL	28.5 (7.3-94.9)	11.4 (4.3-37.6)	<0.001	20.0 (6.6-85.6)	12.8 (5.1-39.9)	<0.001
Peak troponin-I, ng/mL	8.6 (1.7-26.9)	4.4 (1.2-16.1)	<0.001	6.1 (1.5-22.9)	4.2 (1.2-15.4)	0.002
Blood glucose, mg/dL	156.2 ± 72.3	146.7 ± 57.1	0.001	179.2 ± 93.5	164.7 ± 97.6	0.012
Hs-CRP (mg/dL)	0.96 ± 2.72	3.02 ± 13.5	0.001	1.60 ± 8.07	2.48 ± 12.8	0.348
Serum creatinine (mg/L)	1.16 ± 1.29	1.25 ± 1.55	0.113	1.08 ± 1.33	1.09 ± 0.98	0.842
Total cholesterol, mg/dL	179.2 ± 43.9	173.2 ± 43.3	0.001	182.4 ± 48.6	180.8 ± 47.8	0.604
Triglyceride, mg/L	136.0 ± 116.9	132.0 ± 110.4	0.390	126.9 ± 130.2	121.0 ± 69.5	0.329
HDL cholesterol, mg/L	42.4 ± 11.3	40.4 ± 11.2	<0.001	45.3 ± 12.3	44.3 ± 12.9	0.183
LDL cholesterol, mg/L	113.8 ± 38.5	109.3 ± 37.7	0.006	113.8 ± 40.2	114.4 ± 40.6	0.831
GRACE risk score	124.3 ± 40.1	127.9 ± 36.0	0.014	146.7 ± 42.3	148.4 ± 37.7	0.455
>140, *n* (%)	683 (27.4)	273 (32.2)	0.009	408 (49.5)	239 (56.0)	0.032
Discharge medications, *n* (%)						
Aspirin, *n* (%)	2462 (98.8)	832 (98.0)	0.093	815 (98.8)	423 (99.1)	0.782
Clopidogrel, *n* (%)	1720 (69.0)	597 (70.3)	0.491	643 (77.9)	362 (84.8)	0.004
Ticagrelor, *n* (%)	500 (20.1)	160 (18.8)	0.454	144 (17.5)	50 (11.7)	0.008
Prasugrel, *n* (%)	272 (10.9)	92 (10.8)	0.949	38 (4.6)	15 (3.5)	0.459
BBs, *n* (%)	2100 (84.3)	711 (83.7)	0.744	714 (86.5)	352 (82.4)	0.054
ACEI or ARBs, *n* (%)	2032 (81.5)	687 (80.9)	0.683	690 (83.6)	344 (80.6)	0.182
Statin, n (%)	2362 (94.8)	795 (93.6)	0.222	763 (92.5)	398 (93.2)	0.731
Anticoagulant, n (%)	43 (1.7)	23 (2.7)	0.086	14 (1.7)	16 (3.7)	0.031
Infarct-related artery						
Left main, *n* (%)	81 (3.3)	30 (3.5)	0.659	16 (1.9)	19 (4.4)	0.017
LAD, *n* (%)	1045 (41.9)	334 (39.3)	0.213	370 (44.8)	202 (47.3)	0.437
LCx, *n* (%)	666 (26.7)	200 (23.6)	0.070	209 (25.3)	92 (21.5)	0.143
RCA, *n* (%)	700 (28.1)	285 (33.6)	0.002	230 (27.9)	114 (26.7)	0.689
Treated vessel						

Left main, *n* (%)	118 (4.7)	51 (6.0)	0.147	28 (3.4)	26 (6.1)	0.039
LAD, *n* (%)	1385 (55.6)	475 (55.9)	0.873	500 (60.6)	278 (65.1)	0.125
LCx, *n* (%)	976 (39.2)	324 (38.2)	0.625	312 (37.8)	159 (37.2)	0.854
RCA, *n* (%)	914 (36.7)	366 (43.1)	0.001	300 (36.4)	150 (35.1)	0.709
Multivessel disease, *n* (%)	1327 (53.3)	499 (58.8)	0.005	465 (56.4)	250 (58.5)	0.470
ACC/AHA type B2/C lesions, *n* (%)	2100 (84.3)	716 (84.3)	0.964	693 (84.0)	360 (84.3)	0.935
Pre-PCI TIMI flow grade 0/1, *n* (%)	964 (38.7)	338 (39.8)	0.569	315 (38.2)	145 (34.0)	0.155
GP IIb/IIIa inhibitor, *n* (%)	222 (8.9)	77 (9.1)	0.889	66 (8.0)	34 (8.0)	0.982
Transradial approach, *n* (%)	1301 (52.2)	495 (58.3)	0.002	369 (44.7)	219 (51.3)	0.031
IVUS/OCT, *n* (%)	661 (26.5)	233 (27.4)	0.001	170 (20.6)	87 (20.4)	0.941
FFR, *n* (%)	59 (2.4)	26 (3.1)	0.259	15 (1.8)	3 (0.7)	0.138
Drug-eluting stents[a]						
ZES, *n* (%)	629 (25.2)	183 (21.6)	0.033	200 (24.2)	98 (23.0)	0.625
EES, *n* (%)	1313 (52.7)	453 (53.4)	0.780	437 (53.0)	230 (53.9)	0.766
BES, *n* (%)	489 (19.6)	198 (23.3)	0.024	166 (20.1)	88 (20.6)	0.882
Others, *n* (%)	61 (2.4)	15 (1.8)	0.287	22 (2.7)	11 (2.6)	0.924
Stent diameter (mm)	3.11 ± 0.43	3.11 ± 0.43	0.955	2.98 ± 0.39	2.97 ± 0.39	0.559
Stent length (mm)	29.2 ± 13.7	30.3 ± 15.4	0.084	30.2 ± 14.1	29.9 ± 14.1	0.583
Number of stents	1.19 ± 0.45	1.22 ± 0.47	0.176	1.22 ± 0.46	1.21 ± 0.47	0.890

Values are means ± standard deviation or median (interquartile range) or numbers and percentages. The *p* values for continuous data were obtained from the unpaired t-test. The *p* values for categorical data were obtained from the chi-square or Fisher's exact test. LVEF, left ventricular ejection fraction; BMI, body mass index; SBP, systolic blood pressure; DBP, diastolic blood pressure; SDT, symptom-to-door time; DBT, door-to-balloon time; CPR, cardiopulmonary resuscitation; EKG, electrocardiogram; EMS, emergency medical service; PCI, percutaneous coronary intervention; MI, myocardial infarction; CABG, coronary artery bypass graft; HF, heart failure; CK-MB, creatine kinase myocardial band; Hs-CRP, high sensitivity-c-reactive protein; HDL, high-density lipoprotein; LDL, low-density lipoprotein; GRACE, Global Registry of Acute Coronary Events; BBs, beta-blockers; ACEIs, angiotensin converting enzyme inhibitors; ARBs, angiotensin receptor blockers; LAD, left anterior descending coronary artery; LCx, left circumflex coronary artery; RCA, right coronary artery; ACC/AHA, American College of Cardiology/American Heart Association; TIMI, Thrombolysis In Myocardial Infarction; GP, glycoprotein; IVUS, intravascular ultrasound; OCT, optical coherence tomography; FFR, fractional flow reserve; ZES, zotarolimus-eluting stent; EES, everolimus-eluting stent; BES, biolimus-eluting stent. [a] Drug-eluting stents were composed of ZES (Resolute integrity stent; Medtronic, Inc., Minneapolis, MN), EES (Xience Prime stent, Abbott Vascular, Santa Clara, CA; or Promus Element stent, Boston Scientific, Natick, MA), and BES (BioMatrix Flex stent, Biosensors International, Morges, Switzerland; or Nobori stent, Terumo Corporation, Tokyo, Japan).

Table S5. Baseline characteristics between the SDT <24 h and SDT ≥24 h groups before and after PSM.

Variables	Entire patients, *n* = 4593			PSM patients, *n* = 2340			
	SDT <24 h (*n* = 3317, group A + B)	SDT ≥24 h (*n* = 1276, group C + D)	*p* value	SDT <24 h (*n* = 1170)	SDT ≥24 h (*n* = 1170)	*p* value	SD
Age, years	63.4 ± 12.0	66.5 ± 11.9	<0.001	66.5 ± 11.3	65.8 ± 11.9	0.662	-0.60
LVEF, %	54.6 ± 10.2	53.5 ± 11.3	0.002	54.1 ± 10.8	54.1 ± 11.0	0.986	0.01
BMI, kg/m^2	24.1 ± 3.3	24.0 ± 3.4	0.167	23.9 ± 3.2	24.1 ± 3.4	0.337	-0.60
SBP, mmHg	136.7 ± 26.8	133.5 ± 24.1	<0.001	134.7 ± 25.1	134.1 ± 24.2	0.507	0.24
DBP, mmHg	81.9 ± 1585	80.2 ± 14.3	<0.001	80.4 ± 14.3	80.6 ± 14.4	0.733	-0.10
DBT, hours	13.2 (3.9-25.1)	16.5 (4.0-25.8)	0.002	15.5 (4.4-28.2)	16.3 (3.92-25.5)	0.843	-0.03
Cardiogenic shock, *n* (%)	66 (2.0)	15 (1.2)	0.061	11 (0.9)	14 (1.2)	0.688	-0.29
CPR on admission, *n* (%)	108 (3.3)	34 (2.7)	0.341	30 (2.6)	33 (2.8)	0.799	-0.12
Atypical chest pain, *n* (%)	452 (13.6)	296 (23.2)	<0.001	223 (19.1)	227 (19.4)	0.875	-0.07
Dyspnea, *n* (%)	755 (22.8)	374 (29.3)	<0.001	308 (26.3)	311 (26.8)	0.925	-0.01
EKG on admission							
Q-wave, *n* (%)	229 (6.9)	133 (10.4)	<0.001	105 (9.0)	106 (9.1)	0.942	-0.03
ST-segment depression, *n* (%)	779 (23.5)	244 (19.1)	0.001	230 (19.7)	233 (19.9)	0.917	-0.05
T-wave inversion, *n* (%)	716 (21.6)	350 (27.4)	<0.001	330 (28.2)	313 (26.8)	0.459	0.31
Atrial fibrillation, *n* (%)	135 (4.1)	51 (4.0)	0.910	45 (3.8)	46 (3.9)	0.915	-0.05
Killip class 1I/III, *n* (%)	487 (14.7)	241 (18.9)	0.001	205 (17.5)	207 (17.7)	0.957	-0.03
First medical contact							
EMS, *n* (%)	422 (12.7)	46 (3.6)	<0.001	38 (3.2)	46 (3.9)	0.437	-0.38
Non-PCI center, *n* (%)	1677 (50.6)	749 (58.7)	<0.001	688 (58.8)	680 (59.1)	0.769	-0.06
PCI center, *n* (%)	1218 (36.7)	481 (37.7)	0.539	444 (37.9)	443 (37.9)	0.966	0.01
Hypertension, *n* (%)	1725 (52.0)	727 (57.0)	0.003	671 (57.4)	653 (55.8)	0.478	0.32
Diabetes mellitus, *n* (%)	969 (29.2)	448 (35.1)	<0.001	411 (35.1)	398 (34.0)	0.602	0.23
Dyslipidemia, *n* (%)	400 (12.1)	148 (11.6)	0.685	133 (11.4)	141 (12.1)	0.653	-0.22
Previous MI, *n* (%)	227 (6.8)	93 (7.3)	0.605	83 (7.1)	81 (6.9)	0.935	0.07
Previous PCI, *n* (%)	345 (10.4)	118 (9.2)	0.251	124 (10.6)	111 (9.5)	0.409	0.36

Previous CABG, *n* (%)	25 (0.8)	11 (0.9)	0.710	7 (0.6)	9 (0.8)	0.803	-0.24
Previous HF, *n* (%)	49 (1.5)	20 (1.6)	0.788	15 (1.3)	18 (1.5)	0.726	-0.17
Previous stroke, *n* (%)	189 (5.7)	89 (7.0)	0.112	84 (7.2)	78 (6.7)	0.684	0.19
Current smokers, *n* (%)	1264 (38.1)	381 (29.9)	<0.001	350 (29.9)	366 (31.3)	0.501	-0.30
Peak CK-MB, mg/dL	27.0 (7.2-91.8)	12.2 (4.6-41.9)	<0.001	17.0 (5.4-60.3)	12.2 (4.7-43.6)	0.777	0.10
Peak troponin-I, ng/mL	10.5 (2.1-23.4)	5.2 (1.3-22.0)	<0.001	6.1 (1.5-22.9)	5.2 (1.3-22.5)	0.929	0.01
Blood glucose, mg/dL	161.9 ± 77.8	153.0 ± 77.4	0.001	155.7 ± 70.8	153.5 ± 78.6	0.490	0.29
Hs-CRP (mg/dL)	1.29 ± 3.60	2.27 ± 3.25	0.001	1.46 ± 2.80	1.61 ± 2.33	0.134	-0.50
Serum creatinine (mg/L)	1.14 ± 1.30	1.20 ± 1.38	0.180	1.15 ± 1.33	1.19 ± 1.41	0.486	-0.30
Total cholesterol, mg/dL	179.9 ± 44.3	175.8 ± 44.3	0.005	175.5 ± 45.6	175.9 ± 44.5	0.797	-0.08
Triglyceride, mg/L	133.6 ± 116.0	128.7 ± 94.0	0.137	125.0 ± 98.4	130.4 ± 97.0	0.179	-0.50
HDL cholesterol, mg/L	43.1 ± 11.3	41.8 ± 11.4	0.001	42.4 ± 11.2	41.9 ± 11.4	0.376	0.44
LDL cholesterol, mg/L	113.7 ± 36.6	111.3 ± 36.0	0.043	111.4 ± 36.8	111.2 ± 36.3	0.912	0.05
GRACE risk score	129.8 ± 41.8	134.8 ± 37.8	<0.001	133.8 ± 37.5	133.2 ± 38.0	0.714	0.15
>140, *n* (%)	1091 (32.9)	512 (40.1)	<0.001	457 (39.1)	444 (37.9)	0.610	0.25
Discharge medications, *n* (%)							
Aspirin, *n* (%)	3227 (98.8)	1255 (98.4)	0.251	1154 (98.6)	1155 (98.7)	0.857	-0.09
Clopidogrel, *n* (%)	2363 (71.2)	959 (75.2)	0.008	877 (75.0)	871 (74.4)	0.812	0.13
Ticagrelor, *n* (%)	644 (19.4)	210 (16.5)	0.022	190 (16.2)	203 (17.4)	0.507	-0.32
Prasugrel, *n* (%)	310 (9.3)	107 (8.4)	0.330	102 (8.7)	101 (8.6)	0.941	0.03
BBs, *n* (%)	2814 (84.8)	1063 (83.3)	0.204	999 (85.4)	983 (84.0)	0.389	0.38
ACEI or ARBs, *n* (%)	2722 (82.1)	1031 (80.8)	0.327	955 (81.6)	949 (81.1)	0.791	0.12
Statin, n (%)	3125 (94.2)	1193 (93.5)	0.367	1085 (92.7)	1095 (93.6)	0.461	-0.35
Anticoagulant, n (%)	57 (1.7)	39 (3.1)	0.008	33 (2.8)	29 (2.5)	0.700	0.18
Infarct-related artery							
Left main, *n* (%)	97 (2.9)	49 (3.8)	0.133	40 (3.4)	38 (3.2)	0.908	0.11
LAD, *n* (%)	1415 (42.7)	536 (42.0)	0.701	474 (40.5)	492 (42.1)	0.475	-0.32
LCx, *n* (%)	875 (26.4)	292 (22.9)	0.015	298 (25.5)	282 (24.1)	0.473	0.32
RCA, *n* (%)	930 (28.0)	399 (31.3)	0.032	357 (30.5)	358 (30.6)	0.964	-0.02
Treated vessel							

Left main, *n* (%)	146 (4.4)	77 (6.0)	0.026	63 (5.4)	60 (5.1)	0.853	0.13
LAD, *n* (%)	1885 (56.8)	753 (59.0)	0.183	664 (56.8)	690 (59.0)	0.295	-0.44
LCx, *n* (%)	1288 (38.8)	483 (37.9)	0.565	470 (40.2)	447 (38.2)	0.352	-0.41
RCA, *n* (%)	1214 (36.6)	516 (40.4)	0.017	456 (39.0)	466 (39.8)	0.703	-0.16
Multivessel disease, *n* (%)	1792 (54.0)	749 (58.7)	0.004	674 (57.6)	672 (57.4)	0.967	0.04
ACC/AHA type B2/C lesions, *n* (%)	2793 (84.2)	1076 (84.3)	0.964	981 (83.8)	987 (84.4)	0.777	-0.16
Pre-PCI TIMI flow grade 0/1, *n* (%)	1279 (38.6)	483 (37.9)	0.684	425 (36.3)	431 (36.8)	0.830	-0.10
GP IIb/IIIa inhibitor, *n* (%)	288 (8.9)	111 (8.7)	0.986	93 (7.9)	95 (8.1)	0.939	-0.07
Transradial approach, *n* (%)	1670 (50.3)	714 (56.0)	0.001	648 (55.4)	647 (55.3)	0.967	0.02
IVUS/OCT, *n* (%)	831 (25.1)	320 (25.1)	0.986	263 (22.5)	281 (24.0)	0.405	-0.35
FFR, *n* (%)	74 (2.2)	29 (2.3)	0.882	32 (2.7)	28 (2.4)	0.695	0.19
Drug-eluting stents[a]							
ZES, *n* (%)	829 (25.0)	281 (22.0)	0.038	266 (22.7)	265 (22.6)	0.961	0.02
EES, *n* (%)	1750 (52.8)	683 (53.5)	0.670	608 (52.0)	617 (52.7)	0.741	-0.14
BES, *n* (%)	655 (19.7)	286 (22.4)	0.870	275 (23.5)	263 (22.5)	0.589	0.23
Others, *n* (%)	83 (2.5)	26 (2.0)	0.388	21 (1.8)	25 (2.1)	0.656	-0.21
Stent diameter (mm)	3.08 ± 0.42	3.06 ± 0.42	0.235	3.06 ± 0.42	3.07 ± 0.42	0.790	-0.23
Stent length (mm)	29.5 ± 13.8	30.1 ± 15.0	0.203	29.5 ± 14.2	30.0 ± 15.0	0.450	-0.34
Number of stents	1.20 ± 0.45	1.22 ± 0.47	0.256	1.20 ± 0.45	1.22 ± 0.47	0.418	-0.43

Values are means ± standard deviation or median (interquartile range) or numbers and percentages. The *p* values for continuous data were obtained from the unpaired t-test. The *p* values for categorical data were obtained from the chi-square or Fisher's exact test. LVEF, left ventricular ejection fraction; BMI, body mass index; SBP, systolic blood pressure; DBP, diastolic blood pressure; SDT, symptom-to-door time; DBT, door-to-balloon time; CPR, cardiopulmonary resuscitation; EKG, electrocardiogram; EMS, emergency medical service; PCI, percutaneous coronary intervention; MI, myocardial infarction; CABG, coronary artery bypass graft; HF, heart failure; CK-MB, creatine kinase myocardial band; Hs-CRP, high sensitivity-c-reactive protein; HDL, high-density lipoprotein; LDL, low-density lipoprotein; GRACE, Global Registry of Acute Coronary Events; BBs, beta-blockers; ACEIs, angiotensin converting enzyme inhibitors; ARBs, angiotensin receptor blockers; LAD, left anterior descending coronary artery; LCx, left circumflex coronary artery; RCA, right coronary artery; ACC/AHA, American College of Cardiology/American Heart Association; TIMI, Thrombolysis In Myocardial Infarction; GP, glycoprotein; IVUS, intravascular ultrasound; OCT, optical coherence tomography; FFR, fractional flow reserve; ZES, zotarolimus-eluting stent; EES, everolimus-eluting stent; BES, biolimus-eluting stent. [a] Drug-eluting stents were composed of ZES (Resolute integrity stent; Medtronic, Inc., Minneapolis, MN), EES (Xience Prime stent, Abbott Vascular, Santa Clara, CA; or Promus Element stent, Boston Scientific, Natick, MA), and BES (BioMatrix Flex stent, Biosensors International, Morges, Switzerland; or Nobori stent, Terumo Corporation, Tokyo, Japan).

Table S6. Independent predictors for MACCE.

	SDT <24 h				SDT ≥24 h				Total			
	Unadjusted		Adjusted		Unadjusted		Adjusted		Unadjusted		Adjusted	
**Variables**	**HR (95% CI)**	***p* value**	**HR (95% CI)**	***p* value**	**HR (95% CI)**	***p* value**	**HR (95% CI)**	***p* value**	**HR (95% CI)**	***p* value**	**HR (95% CI)**	***p* value**
Male vs. Female	1.783 (0.652 - 0.941)	0.009	1.210 (1.001 - 1.421)	0.020	0.971 (0.736 - 1.281)	0.835	1.309 (0.969 - 1.767)	0.087	0.837 (0.719 - 0.976)	0.023	1.189 (0.999 - 1.379)	0.024
Age, ≥65 years	2.031 (1.708 - 2.415)	<0.001	1.283 (1.042 - 1.580)	0.019	1.961 (1.458 - 2.637)	<0.001	1.549 (1.100 - 2.181)	0.012	2.006 (1.729 - 2.328)	<0.001	1.352 (1.133 - 1.613)	0.001
LVEF, <50%	1.808 (1.521 - 2.150)	<0.001	1.261 (1.048 - 1.519)	0.014	2.451 (1.884 - 3.189)	<0.001	1.898 (1.430 - 2.519)	<0.001	1.980 (1.715 - 2.285)	<0.001	1.429 (1.225 - 1.667)	<0.001
Hypertension	1.571 (1.323 - 1.865)	<0.001	1.210 (1.008 - 1.454)	0.098	1.409 (1.070 - 1.854)	0.015	1.121 (0.832 - 1.512)	0.452	1.525 (1.319 - 1.765)	<0.001	1.091 (1.019 - 1.392)	0.218
Diabetes mellitus	1.835 (1.549 - 2.173)	<0.001	1.440 (1.206 - 1.720)	<0.001	1.620 (1.244 - 2.110)	<0.001	1.288 (0.972 - 1.708)	0.078	1.770 (1.535 - 2.041)	<0.001	1.394 (1.200 - 1.619)	<0.001
Dyslipidemia	1.489 (1.107 - 2.002)	0.008	1.416 (1.049 - 1.911)	0.023	1.115 (0.750 - 1.657)	0.591	1.133 (0.753 - 1.705)	0.549	1.271 (1.003 - 1.611)	0.047	1.215 (0.955 - 1.545)	0.112
DBT	1.001 (0.999 - 1.003)	0.215	0.999 (0.997 - 1.001)	0.311	1.000 (0.998 - 1.002)	0.993	0.998 (0.996 - 1.001)	0.176	1.001 (0.999 - 1.002)	0.385	0.999 (0.997 - 1.000)	0.057
Cardiogenic shock	2.951 (1.976 - 4.408)	<0.001	1.625 (1.234 - 2.019)	0.041	2.281 (0.940 - 5.534)	0.068	2.412 (1.021 - 6.014)	0.021	2.803 (1.946 - 4.037)	<0.001	2.071 (1.607 - 3.149)	0.037
CPR on admission	6.152 (4.704 - 8.045)	<0.001	3.184 (2.372 - 4.275)	<0.001	5.821 (3.636 - 9.319)	<0.001	3.920 (2.368 - 6.489)	<0.001	6.061 (4.802 - 7.652)	<0.001	3.292 (2.558 - 4.235)	<0.001
Atypical chest pain	2.256 (1.856 – 2.741)	<0.001	1.842 (1.508 – 2.251)	<0.001	2.402 (1.835 – 3.143)	<0.001	1.930 (1.459 – 2.553)	<0.001	2.286 (1.965 – 2.672)	<0.001	1.846 (1.571 – 2.619)	<0.001
EMS (+)	1.335 (1.062 - 1.678)	0.013	1.217 (0.981 - 1.535)	0.030	1.954 (1.137 - 3.357)	0.015	1.786 (1.031 - 3.094)	0.039	1.381 (1.121 - 1.703)	0.002	1.401 (1.201 - 1.875)	0.001
GRACE risk score >140	2.738 (2.315 - 3.237)	<0.001	1.751 (1.423 - 2.155)	<0.001	2.227 (1.707 - 2.907)	<0.001	1.675 (1.200 - 2.120)	<0.001	2.579 (2.238 - 2.971)	<0.001	1.633 (1.372 - 1.944)	<0.001
Multivessel disease	1.728 (1.449 - 2.060)	<0.001	1.367 (1.142 - 1.637)	0.001	1.335 (1.013 - 1.758)	0.040	1.112 (0.836 - 1.479)	0.466	1.609 (1.387 - 1.866)	<0.001	1.296 (1.113 - 1.507)	0.003

MACCE, major adverse cardiac and cerebrovascular events; SDT, symptom-to-door time; HR, hazard ratio; CI, confidence interval; LVEF, left ventricular ejection fraction; DBT, door-to-balloon time; CPR, cardiopulmonary resuscitation; EMS, emergency medical service; GRACE, Global Registry of Acute Coronary Events.

Table S7. Independent predictors for all-cause death.

	SDT <24 h				SDT ≥24 h				Total			
	Unadjusted		Adjusted		Unadjusted		Adjusted		Unadjusted		Adjusted	
**Variables**	**HR (95% CI)**	***p* value**	**HR (95% CI)**	***p* value**	**HR (95% CI)**	***p* value**	**HR (95% CI)**	***p* value**	**HR (95% CI)**	***p* value**	**HR (95% CI)**	***p* value**
Male vs. Female	0.631 (0.488 - 0.816)	<0.001	1.400 (1.132 – 2.014)	0.009	0.729 (0.516 – 1.031)	0.074	1.298 (0.874 - 1.762)	0.184	0.649 (0.528 - 0.797)	<0.001	1.387 (1.092 - 1.598)	0.012
Age, ≥65 years	5.090 (3.720 - 6.964)	<0.001	3.051 (1.765 – 5.271)	<0.001	4.648 (2.828 - 7.638)	<0.001	2.237 (1.564 – 3.201)	<0.001	5.035 (3.865 - 6.560)	<0.001	2.514 (1.864 - 3.389)	<0.001
LVEF, <50%	3.219 (2.523 - 4.107)	<0.001	2.473 (1.708 – 3.580)	<0.001	3.687 (2.605 - 5.217)	<0.001	1.663 (1.280 - 2.162)	<0.001	3.405 (2.792 - 4.154)	<0.001	1.899 (1.536 - 2.348)	<0.001
Hypertension	1.680 (1.304 - 2.166)	<0.001	1.037 (0.702 - 1.530)	0.856	1.507 (1.052 - 2.158)	0.025	1.002 (0.765 - 1.311)	0.991	1.640 (1.333 - 2.018)	<0.001	1.018 (0.817 - 1.269)	0.875
Diabetes mellitus	2.361 (1.850 - 3.013)	<0.001	1.209 (0.840 - 1.742)	0.307	1.614 (1.148 - 2.270)	0.006	1.632 (1.264 – 2.105)	<0.001	2.108 (1.729 - 2.570)	<0.001	1.489 (1.209 - 1.833)	<0.001
Dyslipidemia	2.462 (1.437 - 4.219)	0.001	1.073 (0.595 - 1.934)	0.815	1.229 (0.694 - 2.179)	0.480	2.067 (1.200 – 3.561)	0.009	1.875 (1.268 - 2.771)	0.002	1.596 (1.075 - 2.370)	0.020
DBT	1.002 (1.000 - 1.003)	0.093	1.000 (0.998 - 1.002)	0.817	1.001 (0.999 - 1.003)	0.177	0.998 (0.996 - 1.001)	0.203	1.002 (1.000 - 1.003)	0.019	0.999 (0.997 - 1.001)	0.212
Cardiogenic shock	4.725 (2.961 - 7.539)	<0.001	3.102 (1.111 – 8.656)	0.031	3.120 (1.153 - 8.440)	0.025	1.562 (0.956 – 2.551)	0.075	4.199 (2.755 - 6.398)	<0.001	1.778 (1.148 - 2.754)	0.010
CPR on admission	13.81 (10.29 - 18.55)	<0.001	4.962 (2.923 – 8.422)	<0.001	9.634 (5.857 - 15.85)	<0.001	4.652 (3.363 - 6.434)	<0.001	12.34 (9.582 - 15.88)	<0.001	4.593 (3.494 - 6.039)	<0.001
Atypical chest pain	3.906 (3.030 – 5.035)	<0.001	2.696 (2.077 – 3.499)	<0.001	3.521 (2.506 – 4.946)	<0.001	2.454 (1.727 – 3.486)	<0.001	3.832 (3.132 – 4.689)	<0.001	2.585 (2.099 – 3.183)	<0.001
EMS (+)	1.655 (1.213 - 2.259)	0.001	1.376 (0.635 – 2.982)	0.418	1.562 (0.730 - 3.343)	0.251	1.204 (0.872 – 1.661)	0.259	1.518 (1.144 - 2.015)	0.004	1.180 (0.882 - 1.579)	0.265
GRACE risk score >140	8.175 (6.081 - 10.99)	<0.001	1.904 (1.229 - 2.952)	0.004	4.368 (2.980 - 6.404)	<0.001	3.605 (2.545 – 5.106)	<0.001	6.695 (5.300 - 8.457)	<0.001	2.887 (2.197 - 3.795)	<0.001
Multivessel disease	1.696 (1.311 - 2.193)	<0.001	1.014 (0.701 - 1.469)	0.940	1.259 (0.884 - 1.794)	0.202	1.092 (0.840 - 1.421)	0.510	1.554 (1.262 - 1.913)	<0.001	1.069 (0.864 - 1.322)	0.539

SDT, symptom-to-door time; HR, hazard ratio; CI, confidence interval; LVEF, left ventricular ejection fraction; DBT, door-to-balloon time; CPR, cardiopulmonary resuscitation; EMS, emergency medical service; GRACE, Global Registry of Acute Coronary Events.

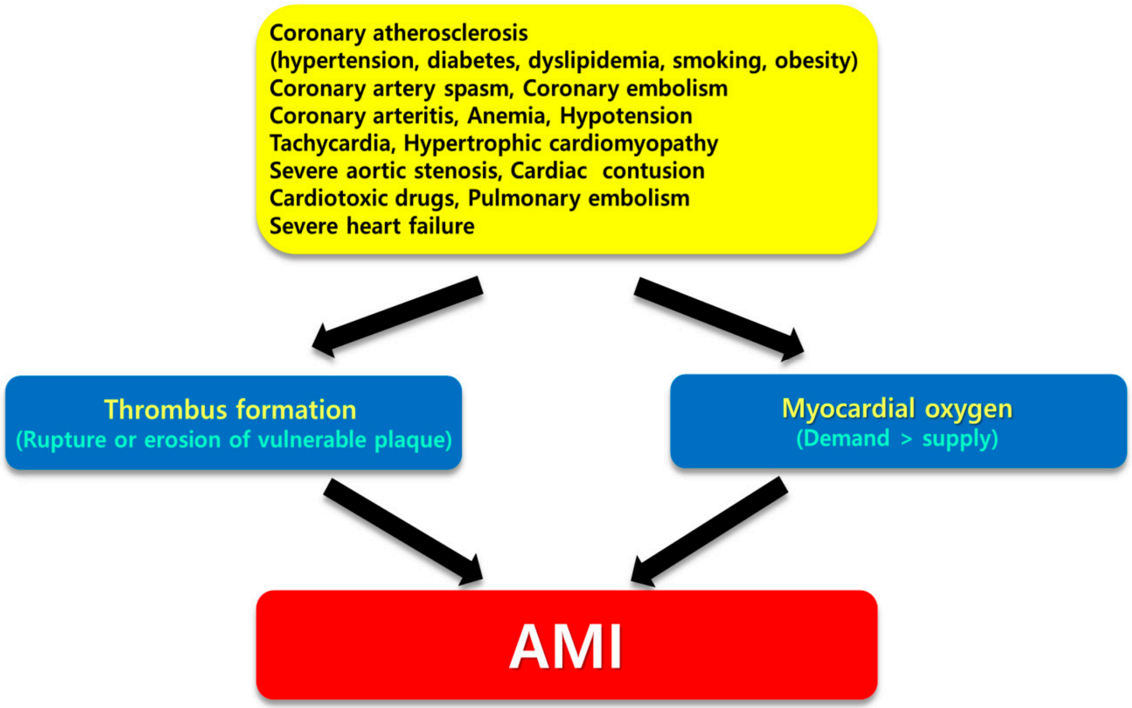

Figure S1. Causes of acute myocardial infarction. AMI, acute myocardial infarction.

SDT <24 h

Subgroup	Male	Female	HR (95% CI)	*p* value
Age ≥65 years	219/951 (23.0%)	132/631 (20.9%)	1.11 (0.89-1.38)	0.347
Age <65 years	173/1541 (11.2%)	29/194 (14.9%)	0.73 (0.49-1.08)	0.116
LVEF <50%	133/597 (22.3%)	69/247 (27.9%)	0.77 (0.57-1.03)	0.074
LVEF ≥50%	259/1895 (13.7%)	92/578 (15.9%)	0.84 (0.66-1.07)	0.156
Atypical chest pain	88/303 (29.0%)	45/149 (30.2%)	0.96 (0.67-1.37)	0.822
Typical chest pain	304/2189 (13.9%)	116/676 (17.2%)	0.79 (0.63-0.98)	0.029
Dyspnea (+)	133/531 (25.0%)	55/224 (24.6%)	1.02 (0.74-1.39)	0.917
Dyspnea (-)	259/1961 (13.2%)	106/601 (17.6%)	0.73 (0.59-0.91)	0.005
Hypertension (+)	226/1163 (19.4%)	117/562 (20.8%)	0.91 (0.73-1.15)	0.452
Hypertension (-)	166/1329 (12.5%)	44/263 (16.7%)	0.73 (0.52-1.01)	0.060
Diabetes mellitus (+)	143/653 (21.9%)	86/316 (27.2%)	0.78 (0.60-1.02)	0.074
Diabetes mellitus (-)	249/1839 (13.5%)	75/509 (14.7%)	0.90 (0.70-1.17)	0.436
GRACE risk score > 140	190/683 (27.8%)	110/408 (27.0%)	1.03 (0.82-1.31)	0.753
GRACE risk score ≤ 140	202/1809 (11.2%)	51/417 (12.2%)	0.90 (0.66-1.23)	0.903

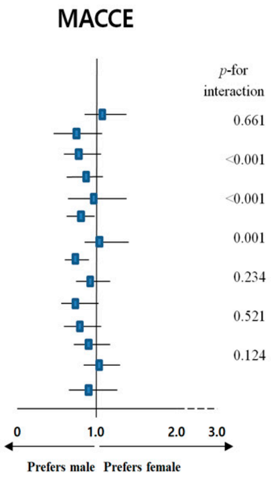

SDT ≥24 h

Subgroup	Male	Female	HR (95% CI)	*p* value
Age ≥65 years	93/411 (22.6%)	69/346 (19.9%)	1.17 (0.86-1.60)	0.321
Age <65 years	53/438 (12.1%)	7/81 (8.6%)	1.42 (0.65-3.12)	0.383
LVEF <50%	71/252 (28.2%)	39/142 (27.5%)	1.06 (0.72-1.57)	0.758
LVEF ≥50%	75/597 (12.6%)	37/285 (13.0%)	0.97 (0.65-1.43)	0.859
Atypical chest pain	55/177 (31.1%)	33/119 (27.7%)	1.15 (0.75-1.78)	0.516
Typical chest pain	91/672 (13.5%)	43/308 (14.0%)	0.99 (0.68-1.41)	0.902
Dyspnea (+)	52/234 (22.2%)	27/140 (19.3%)	1.18 (0.74-1.87)	0.494
Dyspnea (-)	94/615 (15.3%)	49/287 (17.1%)	0.90 (0.63-1.27)	0.532
Hypertension (+)	82/423(19.4%)	61/304 (20.1%)	0.98 (0.70-1.36)	0.888
Hypertension (-)	64/426 (15.0%)	15/123 (12.2%)	1.25 (0.71-2.19)	0.438
Diabetes mellitus (+)	58/275 (21.1%)	43/173 (24.9%)	0.84 (0.57-1.25)	0.391
Diabetes mellitus (-)	88/574 (15.3%)	33/254 (13.0%)	1.20 (0.81-1.80)	0.366
GRACE risk score > 140	72/273 (26.4%)	56/239 (23.4%)	1.17 (0.82-1.65)	0.390
GRACE risk score ≤ 140	74/576 (12.8%)	20/188 (10.6%)	1.24 (0.76-2.03)	0.395

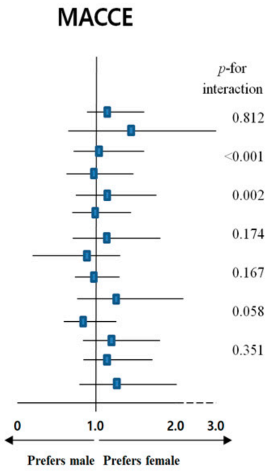

Figure S2. Subgroup analysis for MACCE in the SDT <24 h and SDT ≥24 h groups. MACCE, major adverse cardiac and cerebrovascular events; SDT, symptom-to-door time; HR, hazard ratio; CI, confidence interval; LVEF, left ventricular ejection fraction; GRACE, Global Registry of Acute Coronary Events.

SDT <24 h

Subgroup	Male	Female	HR (95% CI)	*p* value
Age ≥65 years	134/951 (14.1%)	77/631 (12.2%)	1.16 (0.89-1.55)	0.278
Age <65 years	37/1541 (2.4%)	11/194 (5.7%)	0.41 (0.21-0.82)	0.011
LVEF <50%	88/597 (14.7%)	44/247 (17.8%)	0.81(0.57-1.17)	0.260
LVEF ≥50%	83/1895 (4.4%)	44/578 (7.6%)	0.57 (0.39-0.82)	0.002
Atypical chest pain	59/303 (19.5%)	34/149 (22.8%)	0.85 (0.56-1.30)	0.455
Typical chest pain	112/2189 (5.1%)	54/676 (8.0%)	0.63 (0.46-0.87)	0.005
Dyspnea (+)	72/531 (13.6%)	34/224 (15.2%)	0.89 (0.59-1.34)	0.579
Dyspnea (-)	99/1961 (5.0%)	54/601 (9.0%)	0.55 (0.40-0.77)	<0.001
Hypertension (+)	99/1163 (8.5%)	67/562 (11.9%)	0.70 (0.52-0.96)	0.026
Hypertension (-)	72/1329 (5.4%)	21/263 (8.0%)	0.67 (0.41-1.09)	0.104
Diabetes mellitus (+)	77/653 (11.8%)	48/316 (15.2%)	0.77 (0.53-1.10)	0.145
Diabetes mellitus (-)	94/1839 (5.1%)	40/509 (7.9%)	0.64 (0.44-0.93)	0.019
GRACE risk score > 140	127/683 (18.6%)	76/408 (18.6%)	1.01 (0.76-1.34)	0.966
GRACE risk score ≤ 140	44/1809 (2.4%)	12/417 (2.9%)	0.84 (0.45-1.60)	0.603

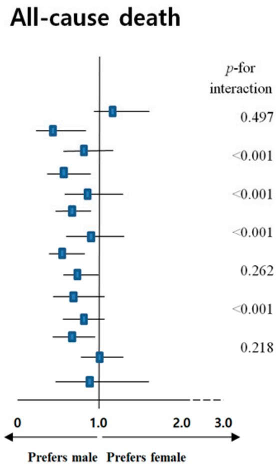

SDT ≥24 h

Subgroup	Male	Female	HR (95% CI)	*p* value
Age ≥65 years	64/411 (15.6%)	51/346 (14.7%)	1.07 (0.74-1.55)	0.705
Age <65 years	15/438 (3.4%)	3/81 (3.7%)	0.92 (0.27-3.20)	0.903
LVEF <50%	51/252 (20.2%)	29/142 (20.4%)	0.99 (0.63-1.57)	0.984
LVEF ≥50%	28/597 (4.7%)	25/285 (8.8%)	0.53 (0.31-0.90)	0.020
Atypical chest pain	38/177 (21.5%)	28/119 (23.5%)	0.91 (0.56-1.48)	0.693
Typical chest pain	41/672 (6.1%)	26/308 (8.4%)	0.72 (0.44-1.18)	0.194
Dyspnea (+)	32/234 (13.7%)	22/140 (15.7%)	0.87 (0.51-1.50)	0.611
Dyspnea (-)	47/615 (7.6%)	32/287 (11.1%)	0.68 (0.43-1.06)	0.091
Hypertension (+)	46/423(10.9%)	42/304 (13.8%)	0.79 (0.52-1.20)	0.265
Hypertension (-)	33/426 (7.7%)	12/123 (9.8%)	0.78 (0.40-1.53)	0.464
Diabetes mellitus (+)	32/275 (11.6%)	29/173 (16.8%)	0.69 (0.42-1.14)	0.146
Diabetes mellitus (-)	47/574 (8.2%)	33/254 (9.8%)	0.83 (0.51-1.34)	0.443
GRACE risk score > 140	52/273 (19.0%)	45/239 (18.8%)	1.03 (0.69-1.54)	0.884
GRACE risk score ≤ 140	27/576 (4.7%)	9/188 (4.8%)	0.98 (0.46-2.09)	0.967

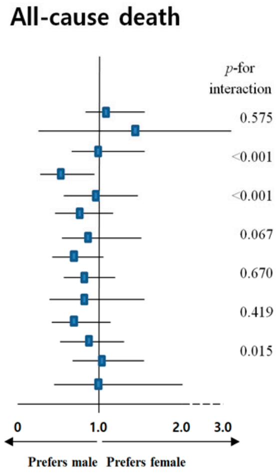

Figure S3. Subgroup analysis for all-cause death in the SDT <24 h and SDT ≥24 h groups. SDT, symptom-to-door time; HR, hazard ratio; CI, confidence interval; LVEF, left ventricular ejection fraction; GRACE, Global Registry of Acute Coronary Events.
